# Engineering Electrospun PVDF Nanofibers for Next-Generation Wearable Bioelectronics

**DOI:** 10.3390/s26196328

**Published:** 2026-10-07

**Authors:** Reshmi Banerjee, Gymama Slaughter

**Affiliations:** 1 Center for Bioelectronics, Old Dominion University, Norfolk, VA 23508, USA; rbanerje@odu.edu; 2 Department of Electrical and Computer Engineering, Old Dominion University, Norfolk, VA 23508, USA

**Keywords:** electrospinning, poly(vinylidene fluoride) (PVDF), piezoelectric nanofibers, wearable bioelectronics, self-powered sensors

## Abstract

Electrospun poly(vinylidene fluoride) (PVDF) and P(VDF-TrFE) nanofibers offer a mechanically flexible, high-surface-area platform for wearable bioelectronics, including piezoelectric sensing and self-powered operation. This review establishes a process–structure–property–performance framework for engineering electrospun PVDF- and P(VDF-TrFE)-based wearable systems. Relationships amongst precursor composition, electrospinning parameters, fiber morphology and alignment, crystalline phase formation, dipole orientation, and device-level piezoelectric performance are examined. Electrospinning is compared with other established fabrication methods including solution casting, spin coating, melt processing, phase inversion, and printing in terms of electroactive β-phase fraction, flexibility, scalability, manufacturing complexity, cost, and wearable-device suitability. Device architectures and performance are evaluated across pressure and strain sensing, physiological monitoring, wound-healing interfaces, piezoelectric and triboelectric nanogenerators, and self-powered wearable sensing applications. Additionally, this review considers the distinction between material-level piezoelectric properties and practical system-level performance, alongside challenges in manufacturing reproducibility, realistic mechanical loading, system integration, and long-term operation. Finally, manufacturing and translational considerations are evaluated, and a conceptual engineering roadmap links material selection and processing with microstructure, device architecture, electronics integration, and translation toward robust, scalable wearable bioelectronic systems.

## 1. Introduction

Wearable bioelectronic systems are increasingly being developed for continuous, noninvasive physiological monitoring by integrating conformal and mechanically flexible sensing, signal acquisition, and data-processing functions. Since Berger’s first scalp electroencephalogram (EEG) recordings in 1929 [1], advances in materials, electronics, fabrication, and device integration have driven the evolution of body-surface physiological monitoring towards flexible and wearable bioelectronic systems [2]. While advances in silicon microelectronics and device miniaturization have enabled the development of compact physiological monitoring systems, conventional semiconductor-based devices are typically mechanically rigid and possess a high Young’s modulus relative to soft biological tissues. The mechanical mismatch at the skin–device interface limits conformal low-impedance contact, which is required for wearable interfaces, despite the mechanical robustness of these devices [3]. Consequently, there is a growing demand for lightweight, flexible, biocompatible, and mechanically compliant materials that are conformal and capable of maintaining stable mechanical and electrical performance at the device–tissue interface to support both sensing and energy harvesting functionalities. For continuous physiological monitoring, wearable devices must be able to withstand repeated mechanical deformation and bending, while maintaining reliable contact and supporting stable signal acquisition. Additionally, depending on the specific sensing modality, they need to either transduce small mechanical inputs, including pressure, strain, vibration, or motion, into measurable electrical signals, or capture low-frequency, low-amplitude electrophysiological signals with a high signal-to-noise ratio for reliable post-processing and analysis [3,4].

Piezoelectric materials are particularly attractive for pressure sensing, strain sensing, vibration sensing, and mechanical energy-harvesting applications, as they are able to directly convert mechanical deformation into electrical output [5,6]. Compared with conventional ceramic piezoelectric materials, PVDF and its copolymers, particularly P(VDF-TrFE), offer a more flexible alternative, combining useful piezoelectric, pyroelectric, and ferroelectric properties with chemical stability, mechanical flexibility, and compatibility with film- and fiber-based fabrication methods [7,8]. Of the various available fabrication techniques, electrospinning is especially relevant due to its ability to produce nanofibrous membranes with high surface area, porosity, low bending stiffness, and mechanical compliance, to promote electroactive β-phase formation and to offer processing advantages associated with fiber- and membrane-level structural control [9,10], making electrospun PVDF and P(VDF-TrFE) particularly relevant to wearable sensing and mechanical energy-harvesting applications.

This review examines the relevance of electrospun PVDF and P(VDF-TrFE) nanofibers to the field of wearable bioelectronics, through a process–structure–property–performance framework, with emphasis on the relationships amongst precursor formulation, electrospinning parameters, fiber morphology and alignment, crystalline-phase development, dipole orientation, device architecture, and experimentally reported sensing and energy-harvesting performance [11,12]. The review further considers key material-, device-, and system-level challenges relevant to wearable implementation and translation.

Previous reviews have established important foundations for understanding PVDF piezoelectric materials and electrospun nanofibers. He et al. [13] specifically examined the effects of electrospinning parameters, including solution, processing, collector, and ambient conditions, on β-phase formation, crystallinity, and piezoelectric properties. Mohammadpourfazeli et al. [14] provided a broader review of PVDF structure, fabrication and modification strategies, electromechanical properties, and applications including energy harvesting, sensing, actuation, and biomedical devices. Building on these studies, the present review focuses specifically on the engineering translation of electrospun PVDF and P(VDF-TrFE) nanofibers for wearable bioelectronics.

To address this objective, the review connects material- and process-level characteristics with device architecture and reported performance. It further compares electrospinning with alternative PVDF fabrication strategies from engineering and manufacturing perspectives, and organizes these considerations within a conceptual roadmap linking material selection and processing to device integration, wearable-system implementation, and eventual clinical or commercial translation.

## 2. PVDF and Piezoelectric Mechanism

PVDF is a semi-crystalline fluoropolymer that has been widely investigated for applications in wearable bioelectronics, actuators, and mechanical energy-harvesting systems because of its lightweight structure, mechanical flexibility, chemical stability, and electroactive behavior. The piezoelectric response of PVDF was first reported by Heiji Kawai in 1969, establishing it as an important piezoelectric polymeric material capable of producing an electromechanical response under mechanical deformation, important for transducer and sensor applications [7]. Since then, PVDF and its copolymer P(VDF-TrFE) (poly(vinylidene fluoride-co-trifluoroethylene)) have been investigated extensively as flexible alternatives to conventional ceramic piezoelectric materials such as lead zirconate titanate (PZT). Since PVDF offers lower density, higher flexibility, easier processability into thin films or fibers, and better mechanical compatibility with soft biological tissues, it is deemed useful as an active material for wearable sensors [8,14,15].

### 2.1. Molecular Structure of PVDF

The electroactive behavior of PVDF originates from its molecular structure and crystalline organization. PVDF consists of repeating –CH_2_–CF_2_– units along a carbon backbone, and the large electronegativity difference between fluorine and hydrogen produces strong local molecular dipoles, primarily associated with the C–F bonds. In each monomer unit, the fluorine atoms draw electron density toward one side of the chain, while the hydrogen atoms form the opposite side of the dipole. However, the presence of molecular dipoles alone does not produce a strong macroscopic piezoelectric response; the dipoles must be organized within a polar crystalline phase and oriented such that their individual moments reinforce rather than cancel one another [8,14,15].

PVDF is a polymorphic polymer that can crystallize into multiple phases (α, β, and γ are most common, while δ and ε phases exist) depending on molecular chain conformation and processing conditions, as shown in Table 1.

**Figure 1:**
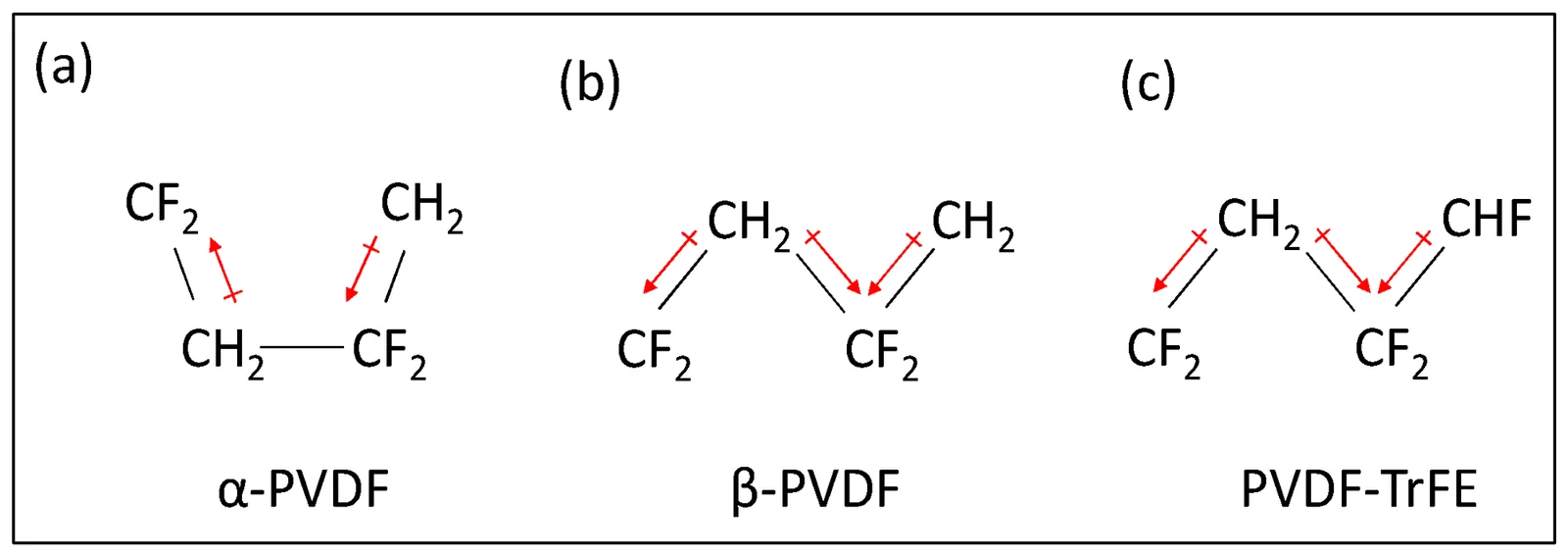
Schematic representation of the PVDF structure illustrating molecular dipole alignment and the resulting net dipole moment: (**a**) α-phase PVDF (μ_net_ ≈ 0); (**b**) β-phase PVDF (μ_net_ ≠ 0); and (**c**) P(VDF-TrFE) (μ_net_ ≠ 0). The local C–F bond dipoles largely cancel in the antiparallel α-phase configuration, resulting in approximately zero net dipole moment, whereas their preferential alignment in β-phase PVDF and P(VDF-TrFE) produces a nonzero net molecular dipole moment.

These phases differ in the arrangement of trans (T) and gauche (G) bonds along the polymer chain, which determines the orientation of molecular dipoles and whether the crystal structure is polar or nonpolar [15]. The α-phase is the most thermodynamically stable phase of PVDF under conventional processing techniques such as melt crystallization or solution casting. It is generally associated with a trans-gauche-trans-gauche′ (TGTG′) chain conformation. Although individual PVDF repeat units contain strong dipoles due to the electronegative fluorine atoms, the α-phase has an antiparallel dipole arrangement within the unit cell. This causes dipole cancellation and results in an overall nonpolar structure [8], as shown in Figure 1 [16,18]. Because of this nonpolar arrangement, α-phase PVDF exhibits limited piezoelectric response unless transformed into an electroactive phase through post-processing (stretching, poling, annealing) techniques.

The β-phase is the most important electroactive phase of PVDF for piezoelectric sensing and energy harvesting. It is characterized by an all-trans (TTTT) chain conformation, in which the polymer backbone adopts a planar zigzag structure. In this conformation, the C–F dipoles are oriented in a more uniform direction, resulting in the highest net dipole moment (Figure 1) among the major PVDF crystalline phases [15].

This strong dipole alignment gives β-phase PVDF its desirable piezoelectric, pyroelectric, and ferroelectric properties. Because of its high polarity, the β-phase is the primary target in PVDF processing for sensors, actuators, and energy-harvesting devices. Several approaches can promote β-phase formation, including mechanical stretching, electrical poling, electrospinning, copolymerization with trifluoroethylene, and incorporation of nucleating fillers [14,15]. Electrospinning is especially effective because the polymer jet experiences strong elongational forces and an electric field during fiber formation. These conditions can align molecular chains, promote dipole orientation, and increase electroactive phase content. This makes electrospun PVDF and P(VDF-TrFE) nanofibers highly relevant for wearable piezoelectric devices [10,17].

The γ-phase is another electroactive phase of PVDF, although it is generally less polar than the β-phase. It is associated with a mixed trans-gauche conformation and can exhibit partial dipole alignment, giving it some electroactive behavior [15]. The γ-phase may form under specific processing conditions, including controlled annealing, solvent processing, crystallization under particular thermal histories, or the use of additives and fillers. While the γ-phase can contribute to the overall electromechanical response, it is usually considered secondary to the β-phase in high-performance piezoelectric sensors and energy harvesters [8,14,15].

### 2.2. Origin of Polarization and Piezoelectric Response

The piezoelectric response of PVDF arises when mechanical deformation changes the polarization state of the material. In a polar PVDF phase, molecular dipoles are arranged to produce a net polarization. When the material is compressed, stretched, bent, or vibrated, deformation alters the polarization, generating an electrical response. PVDF exhibits direct and converse piezoelectric effects. In the direct piezoelectric effect, mechanical deformation generates electrical charge or voltage, whereas the converse effect involves mechanical deformation induced by an applied electric field, enabling actuation. The direct piezoelectric effect is particularly relevant to wearable biosensing and energy harvesting because physiological motion and mechanical inputs such as pressure, strain, vibration, and bending can be converted into measurable electrical output [10,16,17].

The piezoelectric performance of PVDF is closely related to its electroactive crystalline phase composition and the degree of dipole alignment [8,15]. Processing methods play a central role in controlling PVDF phase composition and dipole alignment as they influence piezoelectric response through changes in polarization and structural organization. Mechanical stretching, electrical poling, annealing, copolymerization, filler incorporation, and electrospinning have all been used to increase the electroactive-phase content and/or promote dipole orientation in PVDF-based materials [8,14,15].

Electrical poling is commonly used to align ferroelectric domains and enhance net polarization [8,14], while the electric field and jet stretching during the electrospinning process can promote molecular-chain and dipole orientation. At a larger structural scale, collector-induced fiber alignment can further improve directional load transfer and electromechanical response along the preferred sensing direction [17].

Thus, the piezoelectric behavior of electrospun PVDF-based materials reflects the combined influence of electroactive phase formation, dipole orientation, and fiber organization, providing the basis for evaluating the effects of different fabrication and processing strategies in the following sections.

## 3. Overview of PVDF Fabrication Methods

**Figure 2:**
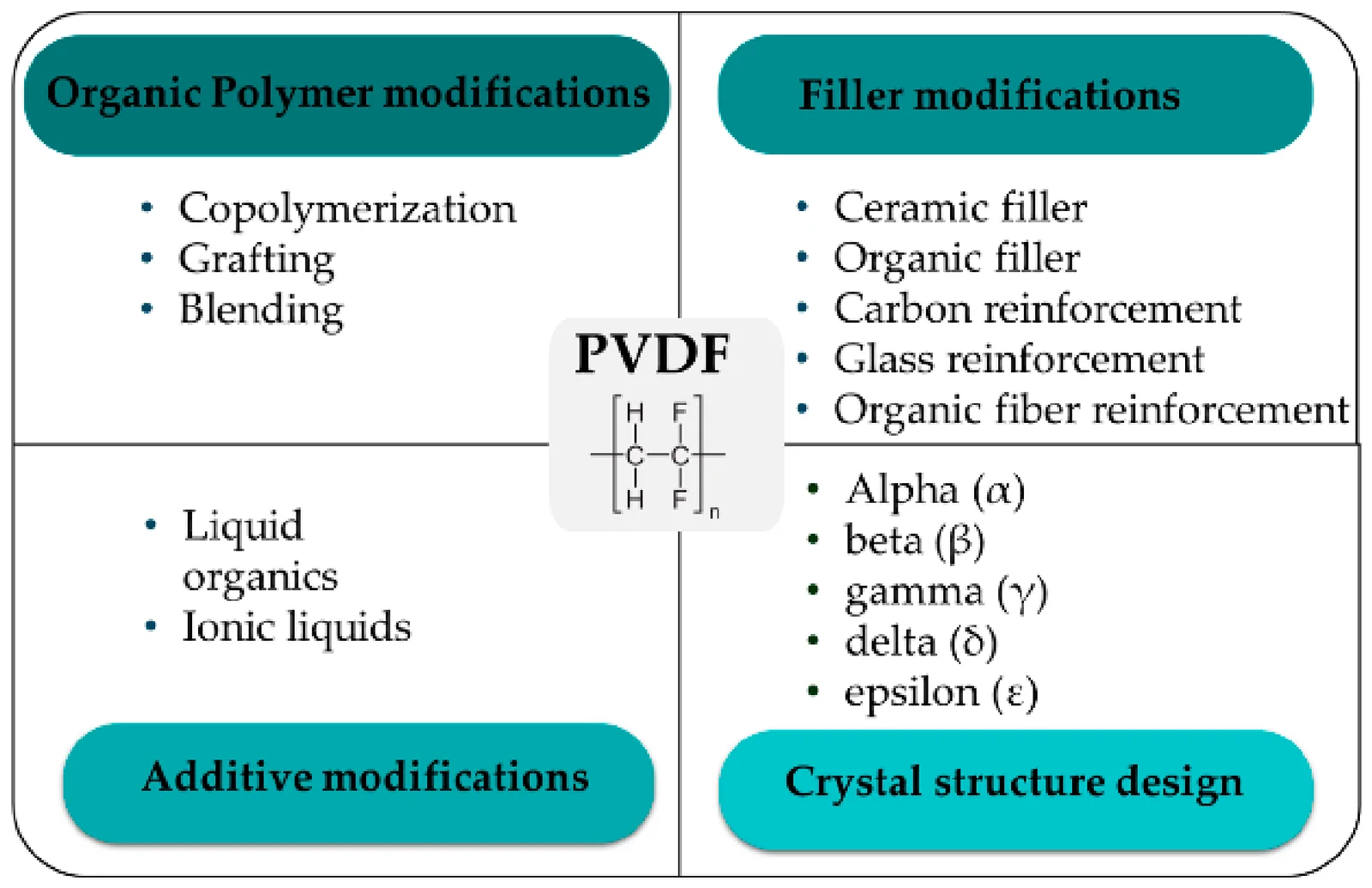
Representative material-modification and structural-design strategies for PVDF, including polymer modification, functional fillers, additives, and crystalline-phase engineering.

As the piezoelectricity of PVDF is strongly dependent on electroactive phase formation and dipole alignment [8,15], the fabrication process serves as the fundamental phase- and structure-engineering strategy, as discussed in Section 3 and Section 4. The fabrication techniques influence film or fiber morphology, crystallinity, electroactive phase formation, mechanical compliance, and device-integration strategy [19]. As summarized in Figure 2 and Table 2, PVDF processing can be carried out using solution casting, spin coating, melt processing, phase inversion, printing, and electrospinning, with each method distinctly influencing the polymer’s crystalline phase, morphology, mechanical properties, device integration, and, most importantly, the piezoelectric response.

Table 2 summarizes the characteristic PVDF structure produced by each fabrication method along with its principal advantages and limitations, to enable a comparison of the various PVDF fabrication routes.

### 3.1. Solution Casting and Drop Casting

Solution casting is one of the simplest PVDF fabrication methods, used for baseline PVDF film formation, wherein the PVDF precursor is dissolved in a suitable solvent or solvent mixture, deposited onto a substrate or mold, and dried to form a film. Solution casting is useful for preparing thin films, composite films, and laboratory-scale samples. It allows straightforward incorporation of fillers or additives and can be used to study phase formation under controlled solvent and drying conditions. However, solution-cast PVDF often requires additional post-processing to enhance piezoelectricity. Depending on solvent type, drying temperature, evaporation rate, and crystallization conditions, solution casting may produce mixtures of α-, β-, and γ-phases. Since the α-phase is commonly obtained under conventional processing, stretching, annealing, electrical poling, or filler-induced nucleation may be required to increase electroactive phase content [15].

### 3.2. Spin Coating

Spin coating is commonly used to fabricate thin and relatively uniform planar PVDF or P(VDF-TrFE) films on flat substrates. A polymer solution is dispensed onto the substrate and spread by centrifugal force during high-speed rotation. The final film thickness depends on solution concentration, spin speed, solvent volatility, and drying conditions. Spin-coated PVDF films are useful for microfabricated devices, capacitors, thin-film sensors, and ferroelectric devices where uniform film thickness is required. The main limitation of spin coating for wearable mechanical sensing is that it produces mechanically flexible planar films rather than porous nanofiber networks, so the resulting films lack the high surface area, porosity, and fiber-level deformability of electrospun mats. Spin-coated films also typically require additional post-processing, such as annealing or poling, to optimize electroactive phase content.

### 3.3. Melt Processing and Compression Molding

Mechanically robust PVDF films can be fabricated through thermoplastic processing methods such as melt extrusion, hot pressing, or compression molding. While melt processing is an attractive fabrication technique for scalability and industrial manufacturing because it avoids solvent use and is compatible with conventional polymer-processing equipment, melt-processed PVDF often crystallizes into phases that may not be optimal for piezoelectric applications unless additional phase transformation steps are applied. Mechanical stretching and electrical poling are commonly needed to enhance β-phase formation and align dipoles. For wearable applications requiring high porosity, low bending stiffness, and large surface area, melt-processed films are found to be less attractive.

### 3.4. Phase Inversion and Membrane Fabrication

Phase inversion is widely used to fabricate flexible, porous PVDF membranes, especially for filtration, separation, and electrochemical applications. In this method, a PVDF solution undergoes solvent–nonsolvent exchange, leading to polymer precipitation and porous membrane formation. Phase inversion can produce mechanically stable porous structures, and the resulting membranes may be useful in applications requiring permeability, fluid transport, or large interfacial area. However, the resulting pore morphology and phase content may not provide superior chain orientation or fiber alignment.

### 3.5. Printing-Based Fabrication

Printing-based approaches, including screen printing, inkjet printing, aerosol-jet printing, and direct writing, are useful for scalable and patterned fabrication of polymer-based devices. PVDF or PVDF-composite inks can potentially be printed into defined patterns, enabling integration with flexible substrates, conductive electrodes, and device arrays. Printing methods are attractive for manufacturing because they allow patterned deposition, reduced material waste, and compatibility with roll-to-roll or large-area processing. The primary challenge with these techniques is that printable PVDF formulations need to satisfy both rheological and electroactive requirements in terms of viscosity, solvent compatibility, drying behavior, adhesion, and phase composition. Printed PVDF layers may also require additional post-treatment such as annealing or electrical poling to enhance electroactive phase content.

### 3.6. Electrospinning

**Figure 3:**
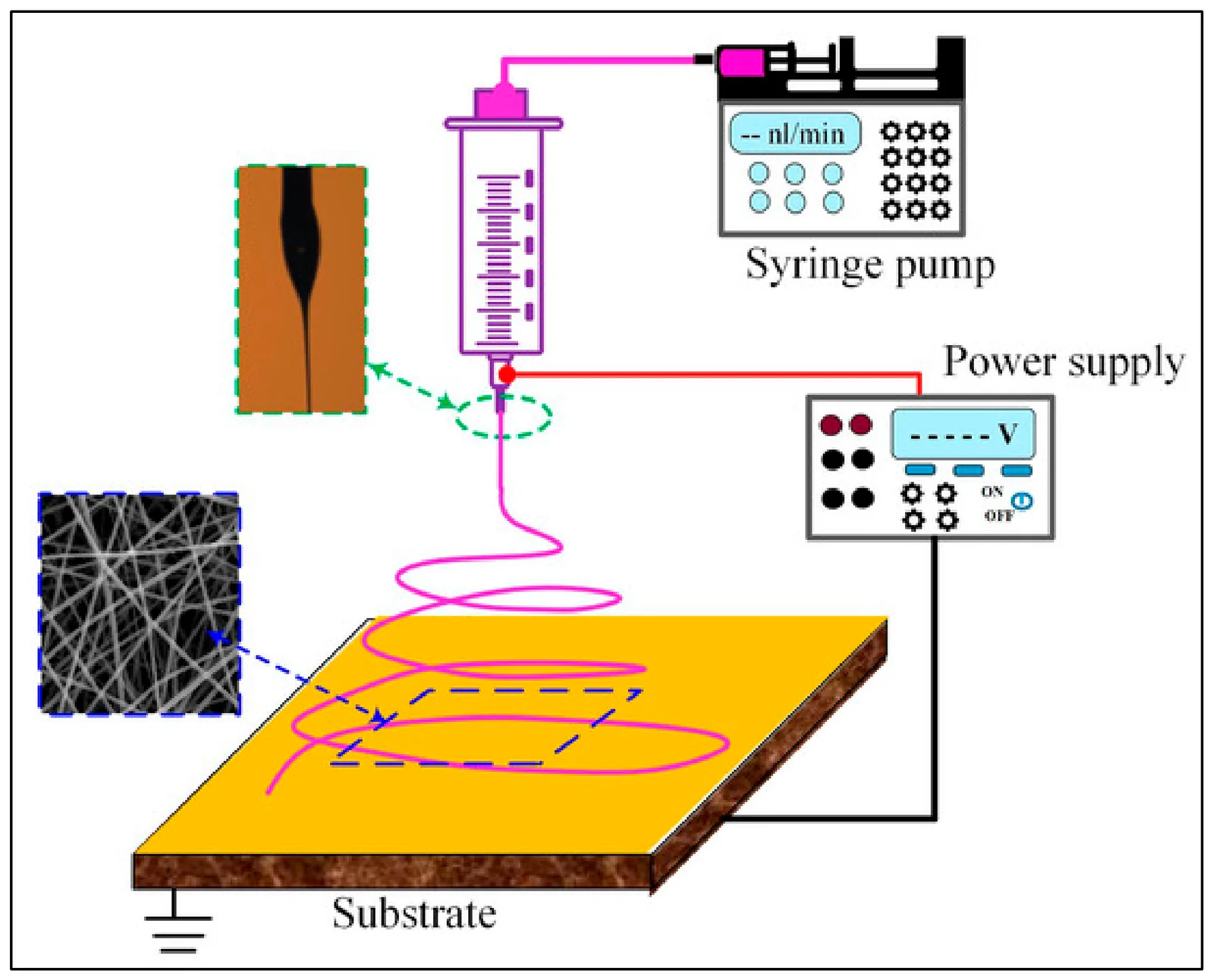
Schematic overview of electrospinning for PVDF nanofiber fabrication.

Electrospinning produces continuous micro- or nanofibrous PVDF membranes with high surface area, porosity, low bending stiffness, and controllable fiber orientation. Compared with conventional film-forming methods, it can simultaneously influence fiber morphology and electroactive-phase development, making it particularly relevant to flexible piezoelectric sensing and energy-harvesting applications [9,13,17,20]. Figure 3 introduce the basic electrospinning configuration, while the electrospinning process details and parameters are discussed in Section 4.

## 4. Electrospinning of PVDF Fibers

Electrospinning is a high-voltage fiber-forming process in which a polymer solution is transformed into continuous fibers through electrostatic forces. A typical electrospinning setup consists of a syringe or reservoir containing the PVDF solution, a metallic needle connected to a high-voltage power supply, a controlled flow-rate system, and a grounded collector. When a sufficiently high electric field is applied, electrostatic forces overcome surface tension at the needle tip and form a Taylor cone. A charged polymer jet is ejected, stretched, and thinned as it travels toward the collector. During this flight, solvent evaporates and solid PVDF fibers are deposited on the collector. The final nanofiber structure is controlled by the interaction of solution parameters, process parameters, collector design, and ambient conditions. Solution parameters include PVDF concentration, molecular weight, solvent system, viscosity, surface tension, and conductivity. Process parameters include applied voltage, flow rate, needle-to-collector distance, and spinning time. Collector parameters include flat plates, rotating drums, parallel electrodes, patterned collectors, and high-speed collectors for aligned fiber formation. Ambient parameters such as humidity and temperature can also affect solvent evaporation, fiber morphology, crystallization, and β-phase formation [13,21].

### 4.1. Process Parameters

Process parameters influence fiber morphology as well as crystallization. The applied voltage must be optimized to balance stable fiber formation with sufficient stretching and dipole alignment, whereas optimization of flow rate and collector distance is necessary for producing uniform PVDF nanofibers with desirable electroactive phase content. The applied voltage controls the electrostatic force that initiates and stretches the polymer jet. A stronger electric field can increase jet elongation and promote molecular chain orientation, which in turn increases the β-phase content. However, excessively high voltage may destabilize the jet, produce bead defects, increase whipping instability, or create nonuniform fiber deposition.

The electric field is important for PVDF because it can influence both morphology and molecular orientation. During electrospinning, the charged jet experiences stretching and polarization forces that can favor electroactive phase formation. Andrew and Clarke showed that electrospinning can directly produce β-phase-rich PVDF fibers from solution, supporting the idea that electrospinning can act as a phase-engineering process rather than only a fiber-forming method [9]. The flow rate system controls the amount of polymer solution supplied to form the Taylor cone. A low flow rate may produce thinner fibers but can interrupt continuous spinning if insufficient solution is delivered, whereas a high flow rate can generate thicker fibers or promote bead formation as the solvent may not evaporate completely before deposition. The needle-to-collector distance affects both jet flight time and solvent evaporation; a comparatively smaller distance would prevent solvent evaporation, whereas unstable jet formation and widespread fiber deposition may be observed at a longer needle-to-collector distance.

### 4.2. Collector Parameters

Collector design strongly influences fiber organization. A stationary flat collector typically produces randomly oriented fiber mats. These mats are easy to fabricate and provide high porosity and large surface area, but their randomly oriented fiber network may lead to less directional electromechanical response. A rotating drum, parallel electrodes, patterned collector, or high-speed collector can be used to produce aligned fiber structures. The influence of collector design on fiber alignment, morphology, and directional mechanical response is demonstrated in the work of Persano et al., where aligned electrospun P(VDF-TrFE) nanofiber arrays form flexible piezoelectric sheets with strong pressure-sensing performance and self-powered mechanical detection [17].

### 4.3. Precursor Material and Formulation

The selection of precursor material and formulation strongly influences the processability, morphology, crystalline-phase development, and piezoelectric response of electrospun PVDF-based nanofibers. Copolymerization of PVDF with fluorinated comonomers such as trifluoroethylene can modify crystallization behavior and improve electroactive phase formation [14,22].

PVDF homopolymer and P(VDF-TrFE) represent two important precursor choices for piezoelectric nanofiber fabrication. PVDF homopolymer is compatible with multiple processing routes, but enhancement of its electroactive β-phase may require phase-engineering strategies such as mechanical stretching, electrical poling, electrospinning, or filler incorporation. P(VDF-TrFE), by contrast, more readily forms polar crystalline conformations because the trifluoroethylene comonomer modifies crystallization behavior [17,22]. Beyond polymer selection, precursor formulation also includes the choice of solvent system and functional additives, which can further influence electrospinnability and the resulting material properties.

#### 4.3.1. Solution Parameters

Molecular weight and polymer concentration strongly influence solution viscosity, chain entanglement, jet stability, fiber diameter, bead formation, and final membrane morphology. Sufficient chain entanglement is required for continuous fiber formation; insufficient polymer concentration can promote bead or droplet formation, whereas excessive concentration can produce high viscosity, unstable flow, thick fibers, or needle clogging. These parameters influence surface texture, fiber morphology, degree of crystallinity, and β-phase development of the resulting PVDF nanofibers [13,21].

Solvent properties affect solution conductivity, viscosity, jet stability, evaporation kinetics, and polymer-chain organization, thereby influencing fiber uniformity, surface morphology, crystallinity, and β-phase development during electrospinning. A high dielectric constant and good solubility can help improve the charge-carrying capacity and jet stability, while volatile solvents can accelerate solidification, and mixed-solvent systems are often used to balance solubility and evaporation rate.

PVDF is commonly dissolved in polar aprotic solvents such as N,N-dimethylformamide (DMF), N,N-dimethylacetamide (DMAc), dimethyl sulfoxide (DMSO), or N-methyl-2-pyrrolidone (NMP), often mixed with more volatile solvents such as acetone to control evaporation rate, jet solidification, and fiber morphology. Mixed-solvent systems are therefore a commonly used strategy to balance polymer solubility, charge transport, and solvent evaporation during fiber formation [13,21].

#### 4.3.2. Role of Functional Additives in Precursor Formulations

Functional additives can be incorporated into the precursor formulation to modify crystalline-phase development, dielectric behavior, mechanical properties, charge transport, and electromechanical response. Nanofillers including carbon nanotubes, graphene, BaTiO_3_, ZnO, clay, and metal nanoparticles have been investigated as modifiers of PVDF-based nanofibers. Their effects may arise through mechanisms including filler-induced β-phase nucleation, interfacial polarization, local electric-field effects, and changes in polymer-chain organization. However, excessive filler loading can increase solution viscosity, promote aggregation and fiber defects, reduce flexibility, or introduce electrical leakage, emphasizing the need to optimize filler concentration and dispersion [13,21].

MXenes have recently emerged as a particularly relevant class of two-dimensional functional fillers for PVDF-based piezoelectric composites. They are a family of transition-metal carbides, nitrides, and carbonitrides, commonly represented as *M_n_*_+1_*X_n_T_n_*, where M is a transition metal and X is carbon and/or nitrogen, while T represents surface functional group terminations such as oxides (–O), hydroxyl groups (–OH) and fluorides (–F). Varying MXene composition and surface terminations enable fine-tuning of the surface chemistry, electrical and mechanical properties, as well as interfacial interactions with polar polymers, making them particularly attractive as a functional modifier in piezoelectric fluoropolymer composites [23].

In PVDF and P(VDF-TrFE) composites, the high electrical conductivity, large interfacial area, and surface functional groups of MXenes can promote interfacial polarization and stabilization of electroactive polymer-chain conformations [24,25]. Surface terminations may additionally facilitate electrostatic interactions and hydrogen bonding with PVDF chains, influencing chain organization and α-to-β phase transformation [26]. In electrospun PVDF/MXene composites, increases in β-phase content, electromechanical response, and charge-transport characteristics have been reported; however, these effects depend strongly on MXene concentration, dispersion, and interfacial compatibility with the polymer matrix [24,25].

Excessive MXene loading may increase electrical leakage, dielectric loss, filler aggregation, and solution viscosity, thereby compromising electrospinnability, fiber uniformity, and mechanical flexibility. Oxidation and restacking of MXene sheets can additionally affect material stability, dispersion, and processing consistency [23,25]. MXene incorporation should therefore be considered a concentration- and interface-dependent composite-engineering strategy, rather than an intrinsically superior route to improved piezoelectric performance [24].

For wearable bioelectronics, precursor formulation therefore requires balancing electrospinnability, electroactive-phase development, mechanical flexibility, biocompatibility, and compatibility with subsequent electrode and device integration. In composite systems, polymer composition, filler concentration, dispersion, and interfacial interactions must be considered together because each can influence fiber morphology, crystalline structure, and electromechanical response.

### 4.4. Structure–Property Relationship

**Figure 4:**
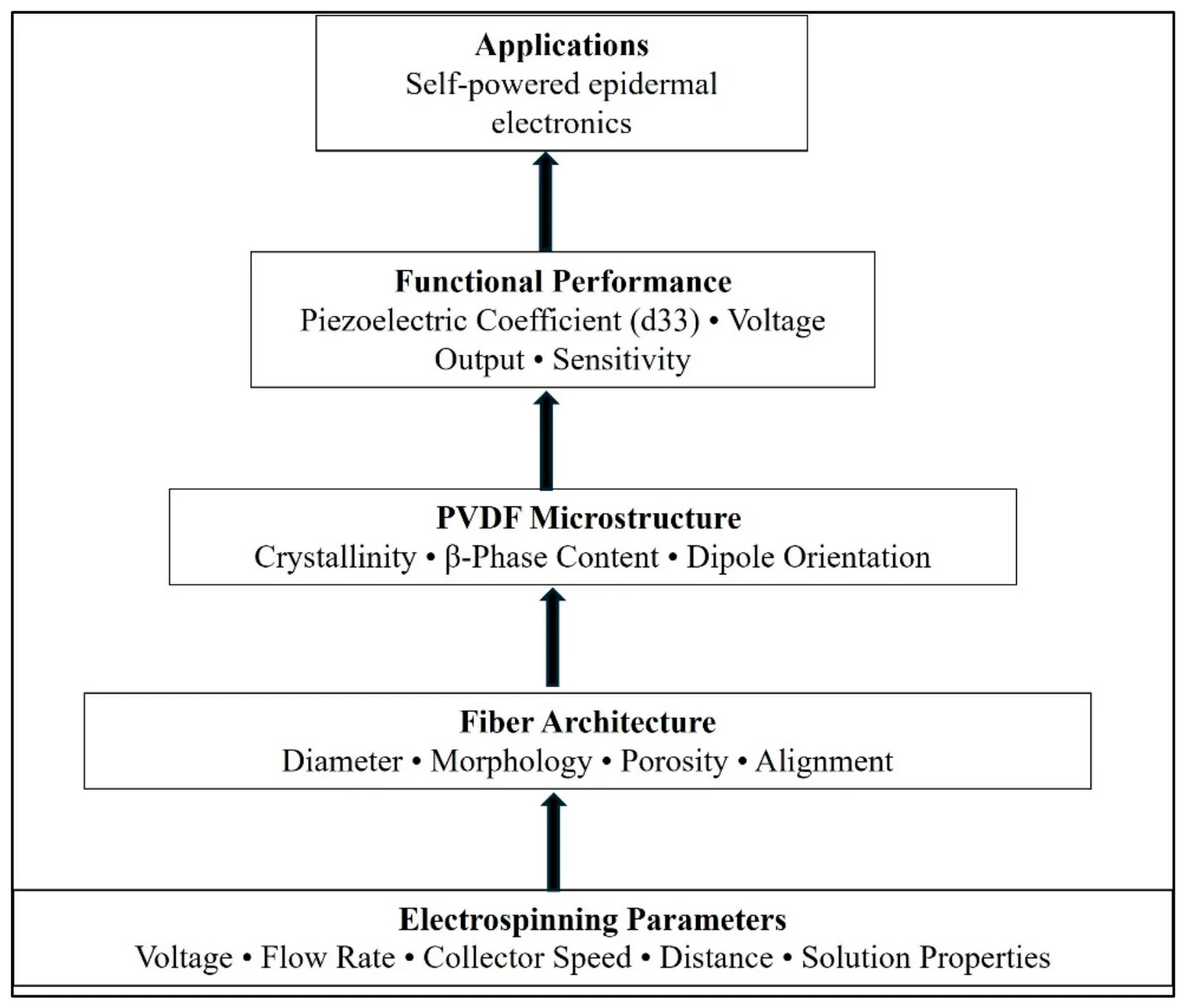
Hierarchical process–structure–property relationship of electrospun PVDF nanofibers.

The structure–property relationship is central to understanding the performance of electrospun PVDF nanofibers. Electrospinning parameters influence fiber architecture, including diameter, morphology, porosity, and alignment, as well as microstructural characteristics such as crystallinity, β-phase content, and dipole orientation. Through the combined effects of the applied electric field and elongational stretching during fiber formation, electrospinning can promote electroactive β-phase development and, in some PVDF systems, reduce the need for additional post-processing such as mechanical stretching or electrical poling [13]. These features collectively affect piezoelectric response and, together with device architecture and loading conditions, determine device-level metrics such as piezoelectric coefficient, voltage output, sensitivity, and sensing or energy-harvesting performance. Optimization therefore requires coordinated control of material formulation, spinning conditions, collector design, and device architecture. The hierarchical relationship among processing conditions, fiber architecture, microstructure, and device-level performance is summarized in Figure 4.

#### 4.4.1. Fiber Alignment and Directional Response

Electrospinning allows control over fiber orientation through collector design. Random fiber mats are useful for flexible and porous sensing layers, while aligned fiber arrays can improve anisotropic mechanical response and directional piezoelectric output. Aligned fibers can also improve load transfer along the fiber direction and promote more organized device architectures. This is especially useful for pressure sensors, strain sensors, vibration sensors, and seismocardiogram (SCG)-relevant mechanical sensing, where the direction and magnitude of mechanical deformation affect signal output [9].

#### 4.4.2. Porosity, Mechanical Compliance, and Wearability

Electrospun nanofiber membranes combine high surface-area-to-volume ratios with porous structures, increasing the deformable active area, improving interaction with electrodes, fillers, coatings, or encapsulation layers, and supporting breathable device architectures. Because the fibrous network can bend, compress, and recover more easily than a dense film, porous nanofiber mats also tend to respond more sensitively to small mechanical deformation [21]. This same porosity also underlies the mechanical compliance of electrospun PVDF mats: unlike dense films, which may resist bending or delaminate under repeated loading, nanofiber mats distribute deformation across the fibrous network, allowing them to conform to curved body surfaces. This combination is particularly valuable for body-worn pressure, vibration, and motion sensors, as well as energy harvesters that must tolerate repeated bending [27].

#### 4.4.3. Piezoelectric Coefficients: *d*_31_ and *d*_33_

One of the most important material-level parameters is the piezoelectric coefficient, commonly represented as *d*_ij_, which describes the electrical charge generated per unit mechanical force or the mechanical strain generated per unit electric field, depending on the measurement mode [5]. Piezoelectric coefficients (*d*_31_ and *d*_33_) are used to describe direction-dependent electromechanical coupling. For the direct piezoelectric effect, the charge coefficient is expressed as charge per unit force (C/N, or pC/N), whereas for the converse effect the strain coefficient is expressed as strain per unit electric field (m/V, or pm/V). The values are presented to illustrate reported processing–structure–property relationships, rather than to establish a quantitative ranking among fabrication methods, as material composition, sample geometry, post-processing conditions, characterization methods, and piezoelectric measurement protocols differ among the cited studies. Table 3 summarizes representative, study-specific β-phase and piezoelectric response data for selected PVDF processing strategies, with direct and converse *d*_33_ measurements presented separately because they arise from different measurement modes and experimental protocols.

The reported β-phase fractions in Table 3 represent study-specific values rather than directly comparable absolute quantities, as the underlying characterization and calculation methods differ across studies. FTIR- and FTIR-ATR-derived β-phase fractions depend on the selected absorption bands, baseline treatment, absorption coefficients, and calculation method used, while XRD, Raman, and DSC may provide complementary or supporting phase characterization rather than an equivalent phase-characterization measure. Additionally, the reported piezoelectric response depends on polymer composition, film or fiber geometry, thickness, poling history, thermal or mechanical post-processing, electrode configuration, and measurement mode. Accordingly, Table 3 illustrates representative processing–property trends rather than providing a direct quantitative ranking of fabrication methods or piezoelectric performance across studies.

In addition to the distinction between direct and converse measurements, the relevant piezoelectric coefficient depends on the device loading mode. While *d*_33_ is most relevant when the mechanical stress and polarization directions are approximately parallel, for through-thickness compression and sandwich-type device configurations, a *d*_31_-type response is associated with mechanical deformation applied along an axis perpendicular to the polarization direction, making it particularly relevant to bending, tensile, and in-plane deformation. Accordingly, the dominant piezoelectric mode in a wearable PVDF device is strongly influenced by device architecture, electrode placement, polarization direction, and the direction of mechanical loading.

### 4.5. Comparative Engineering Assessment of PVDF Fabrication Strategies

Although individual fabrication methods have distinct advantages, selecting an appropriate manufacturing strategy requires balancing piezoelectric performance, mechanical compliance, scalability, and cost. While Table 3 focuses on the quantitative relationship between processing route, β-phase development, and measured piezoelectric response, these metrics alone do not determine suitability for wearable bioelectronics applications; Table 4 and Table 5 provide a qualitative engineering comparison of the fabrication strategies in terms of flexibility, scalability, processing complexity, relative cost, and manufacturing readiness. A descriptive comparative rating is provided in Table 4 based on the operational criteria defined in Table 5 and is intended to support comparative interpretation rather than represent standardized performance scores or formal technology-readiness levels. Each attribute is assessed independently because a fabrication method may perform favorably for one engineering criterion while presenting limitations for another.

Amongst the various fabrication approaches considered in Table 2, Table 3 and Table 4, electrospinning remains especially attractive for wearable bioelectronics because it can simultaneously promote electroactive phase formation and generate flexible, high-surface-area fibrous architectures. However, translation from laboratory-scale demonstrations to scalable manufacturing remains dependent on improvements in throughput, fiber and membrane uniformity, multi-parameter process control, and integration with continuous manufacturing platforms.

Solution casting and melt-based approaches generally offer simpler scale-up pathways with limited control over the desired nanofibrous architecture and may require additional post-processing for enhancement of electroactive properties. Printing and direct-writing methods offer greater patterning and device-integration flexibility, but their scalability and reproducibility depend strongly on control of ink rheology, deposition conditions, drying behavior, and post-processing. Therefore, selection of a specific fabrication strategy is driven by (a) careful evaluation of the comparative tradeoffs associated with each process, along with (b) an appropriate balance of piezoelectric performance, structural flexibility, manufacturability, device integration and, most importantly, the application-specific requirements.

Flexibility reflects the mechanical compliance of the resulting structure; scalability reflects compatibility with large-area, high-throughput, or continuous manufacturing; Process Complexity reflects equipment requirements, process-control burden, and post-processing steps; Cost reflects the relative burden associated with materials, specialized equipment, and processing; and Manufacturing Readiness (Mfg. Readiness) reflects the relative maturity of the process for controlled and scalable device fabrication. Because these attributes are strongly dependent on formulation, equipment, processing parameters, and device architecture, the High (H), Moderate (M), and Low (L) ratings in Table 4 should be interpreted as comparative engineering trends rather than absolute rankings. It is important to note that while higher ratings indicate greater capability for Flexibility, Scalability, and Manufacturing Readiness, higher ratings indicate greater processing or resource burden for the Process Complexity and Cost categories in Table 4 and Table 5.

Beyond the attributes summarized in Table 4, fabrication reproducibility and wearable suitability remain important considerations; however, these attributes are strongly influenced by study-specific processing conditions and device-level integration. Reproducibility is affected by formulation, equipment, environmental conditions, and process control, whereas wearable suitability depends on factors such as conformability, substrate selection, encapsulation, electrode configuration, and attachment strategy. The fabrication techniques discussed in Table 4 can support flexible, mechanically compliant, and relatively thin or conformal device structures to varying degrees; however, their ultimate suitability for wearable, on-skin implementation depends on the specific device design, application requirements, and integration of these device-level factors.

## 5. Applications

Building on the structure–property relationships, this section examines representative device architectures and applications of electrospun PVDF- and P(VDF-TrFE)-based nanofibers, spanning pressure and strain sensing, physiological monitoring, wound healing, and energy harvesting.

### 5.1. PVDF Wearable Device Architectures

The performance of electrospun PVDF- and P(VDF-TrFE)-based wearable devices is governed not only by material composition and electroactive-phase content but also by device architecture and the mechanical loading conditions under which the device operates. Device architecture determines how pressure, strain, bending, vibration, or body motion is transferred to the piezoelectric nanofiber layer, how deformation is distributed within the active material, and how the resulting electrical charge is collected by the electrodes. Consequently, the measured device-level response reflects the combined effects of material properties, fiber organization, device geometry, electrode configuration, mechanical coupling, and loading conditions. The device architecture section, therefore, provides an important link between the structure–property relationships discussed in Section 4 and the wearable sensing and energy-harvesting applications considered in Section 5.3 and Section 5.4.

Free-standing electrospun nanofiber mats represent one of the simplest device configurations. These structures are typically collected as porous fibrous membranes and subsequently integrated with flexible electrodes to form pressure sensors, motion sensors, strain-responsive devices, or piezoelectric nanogenerators. Their porous morphology and low bending stiffness allow the fiber network to undergo compression, bending, tensile deformation, and local rearrangement under relatively small mechanical inputs. Randomly oriented mats can provide multidirectional mechanical compliance because the fibers are distributed over multiple orientations; however, this distribution can also reduce the directional contribution of individual fibers to the net piezoelectric output. Their measured response therefore depends strongly on fiber morphology, membrane thickness, electrode contact, mechanical boundary conditions, and the manner in which the external load is transferred to the fibrous network [20,21].

Fiber alignment provides an alternative architecture when directional mechanical response is required. Aligned PVDF or P(VDF-TrFE) nanofiber arrays can improve anisotropic load transfer and reduce cancellation of piezoelectric contributions when deformation occurs preferentially along the fiber orientation. Aligned electrospun P(VDF-TrFE) arrays have been demonstrated as flexible piezoelectric structures for pressure sensing and self-powered mechanical detection [16,20]. Such architectures are particularly relevant to applications involving directional strain, vibration, and mechanically induced physiological signals. Alignment, however, should not be interpreted independently as an intrinsic measure of superior device performance because the measured response also depends on fiber density, membrane thickness, electrode geometry, loading direction, mechanical constraint, and test configuration.

Sandwich structures are widely used in PVDF-based piezoelectric sensors and nanogenerators. In a typical sandwich device, the electrospun PVDF nanofiber layer is placed between top and bottom electrodes. When mechanical pressure or bending is applied, deformation of the piezoelectric layer generates charge that is collected by the electrodes. This architecture is simple, effective, and compatible with wearable pressure sensors and energy harvesters. Stacked and multilayer structures can further improve performance by increasing active piezoelectric volume, enhancing stress transfer, and enabling composite layer design and control over both the active piezoelectric layer as well as the mechanical/electrical interfaces. Multilayer electrospun structures may include pure PVDF layers, filler-loaded PVDF layers, conductive interlayers, or protective encapsulation layers [20,21].

Core–shell and composite nanofiber architectures provide further control over interfacial, dielectric, conductive, and mechanical properties. In composite fibers, functional fillers are incorporated directly into the PVDF-based matrix, whereas core–shell structures spatially separate materials with different functional roles. These configurations can influence interfacial coupling, dielectric response, charge transport, mechanical reinforcement, and electromechanical behavior. Their effectiveness depends on filler concentration, dispersion, interfacial compatibility, and preservation of stable fiber morphology and mechanical flexibility [20,21]. The material-level role of functional additives and composite formulation is discussed separately in Section 4.3.2.

Electrospun PVDF nanofibers can also be incorporated into textile-, yarn-, and fabric-based architectures to support conformability, breathability, mechanical flexibility, and garment-level integration. In these systems, deformation can arise from combinations of stretching, bending, folding, compression, fiber sliding, and movement between the active material and the textile substrate. Their loading environment therefore differs from that of planar sandwich devices and must be considered when interpreting wearable performance [20,34]. Asymmetric multilayer architectures provide another distinct configuration, particularly for therapeutic and wound-healing bioelectronics. These systems combine a piezoelectric layer with additional components designed for functions such as moisture management, antibacterial activity, tissue protection, or other wound-interface requirements. Their design must therefore balance piezoelectric response with mechanical compliance, tissue contact, biocompatibility, and the additional functional requirements of the therapeutic interface.

### 5.2. Device-Level Metrics

Wearable sensor performance is commonly evaluated using electrical output and sensing metrics including open-circuit voltage, short-circuit current, sensitivity, response or recovery time, signal-to-noise ratio (SNR), stability under cyclic loading, operating pressure range, and mechanical durability. Device-level output also depends on active area, thickness, electrode configuration, mechanical coupling, applied load, loading frequency, encapsulation, and electrical measurement conditions. Wearable sensing performance is therefore commonly characterized using quantities such as voltage output, force or pressure sensitivity, strain sensitivity, detection range, response time, and stability under repeated loading.

Device-level performance depends on both material properties and architecture-dependent mechanical and electrical coupling, which determine how piezoelectric deformation is translated into measurable electrical response. Amongst various metrics, *d*_31_ and *d*_33_ coefficients are particularly relevant for relating device structure to the dominant mechanical loading and charge-collection configuration.

Sandwich structures can favor through-thickness compression and charge collection across the active layer. In such configurations, a *d*_33_-type response is particularly relevant when mechanical stress and polarization are oriented predominantly along the thickness direction. Applied pressure deforms the piezoelectric layer between the electrodes, allowing the resulting charge to be collected across the device. By contrast, free-standing films and nanofiber mats can undergo combinations of tensile, bending, and compressive deformation rather than a purely through-thickness loading mode.

The *d*_31_ coefficient becomes particularly relevant to bending, stretching, and in-plane deformation. In flexible or free-standing structures, bending and tensile deformation can generate a *d*_31_-type response, whereas aligned nanofiber arrays can provide directional mechanical load transfer along the preferred fiber orientation. In this mode of operation, mechanical deformation occurs along one direction while the electrical response is associated with a perpendicular direction. Consequently, the same PVDF-based material may exhibit different effective responses depending on whether the device experiences predominantly compression-dominant *d*_33_ loading, bending/tensile *d*_31_ loading, or mixed deformation. Device architecture therefore influences which piezoelectric response becomes most relevant under a given loading condition.

Stacked and multilayer architectures can further modify active piezoelectric volume and stress distribution, while core–shell and composite nanofibers can alter interfacial polarization, dielectric behavior, charge transport, and electromechanical coupling. Electrospun nanofiber devices also commonly experience mixed mechanical loading, including compression, bending, fiber sliding, and local strain redistribution.

As the device-level metrics are reported using different physical bases across the literature, Table 6 compares representative sensing performance while distinguishing force-, pressure-, and strain-normalized sensitivity and the associated test configurations.

Table 6 illustrates how the electromechanical behavior of electrospun PVDF-based materials is translated into measurable device-level performance under different loading and test conditions. The reported force, pressure, and strain sensitivities, together with output voltage and device geometry, reflect not only the intrinsic piezoelectric response of the active material but also the direction and magnitude of mechanical deformation, electrode configuration, and mechanical coupling within the device. These factors directly influence whether *d*_33_- or *d*_31_-type behavior is most relevant to the measured response, thereby relating the device-level metrics summarized in Table 6 to the architecture- and loading-dependent piezoelectric behavior discussed above.

Device-level measurements should be interpreted in relation to the architecture and loading configuration used in each study. Electrospun nanofiber devices often experience mixed mechanical loading, including compression, bending, tensile strain, fiber sliding, and local strain redistribution. Consequently, reported voltage output, sensitivity, current, and power density should be interpreted together with device geometry, mechanical stress transfer, electrode placement, loading magnitude and frequency, and charge-collection configuration because these factors can substantially influence the measured device-level response.

**Figure 5:**
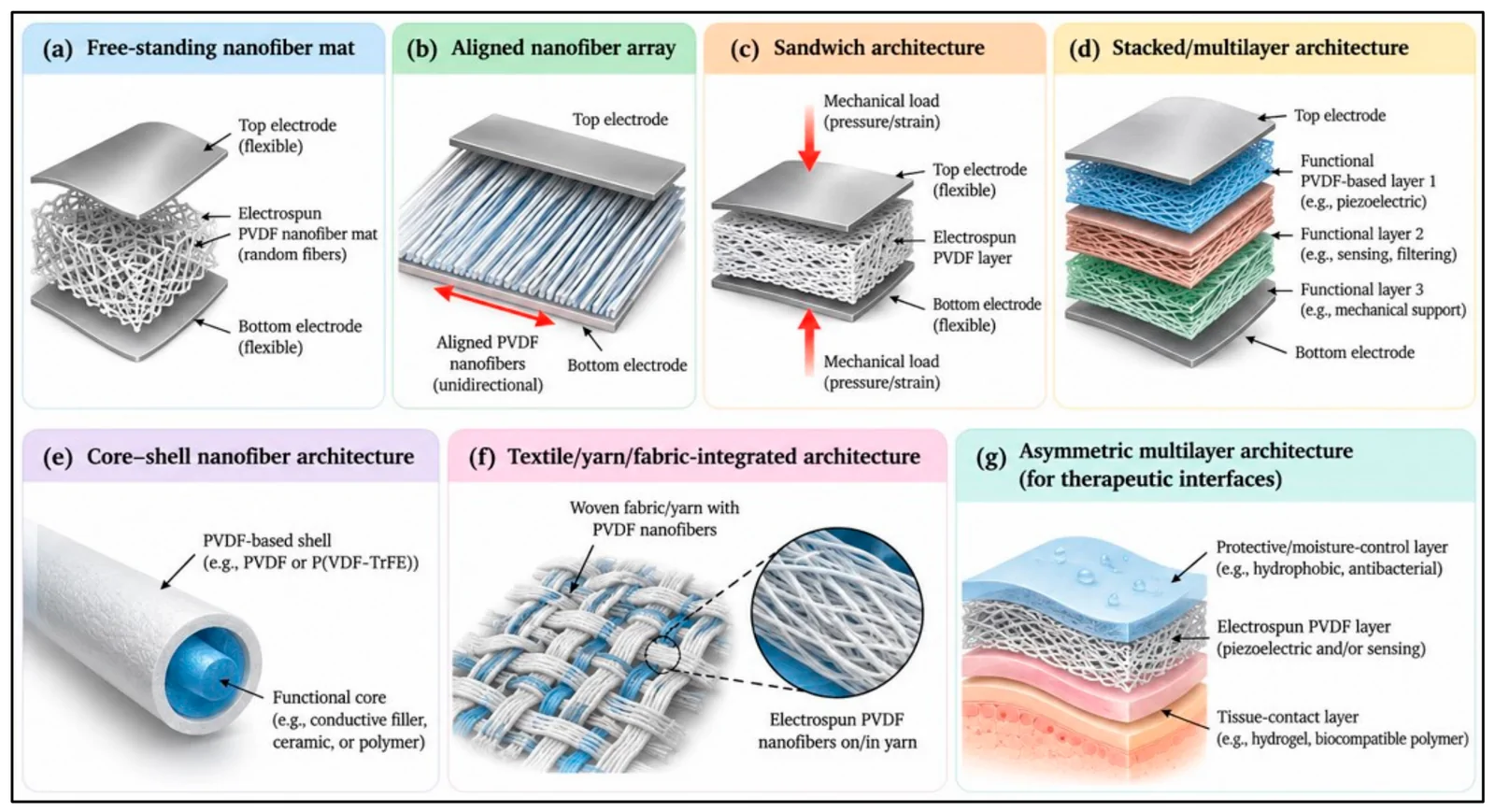
Representative device architectures of electrospun PVDF-based wearable bioelectronics: (**a**) random nanofiber mat reproduced from [41]; (**b**) aligned nanofiber array reproduced from [17]; (**c**) sandwich architecture reproduced from [41]; (**d**) stacked/multilayer architecture reproduced from [42]; (**e**) core–shell nanofiber architecture reproduced from [43]; (**f**) textile/yarn/fabric-integrated architecture reproduced from [34]; and (**g**) asymmetric multilayer architecture for therapeutic interfaces reproduced from [44].

Reported voltage output, sensitivity, current, and power density should therefore be interpreted together with device architecture and test configuration because mechanical stress transfer, deformation mode, electrode placement, loading conditions, and charge collection can substantially influence the measured device-level response. As shown in Figure 5, electrospun PVDF- and P(VDF-TrFE)-based nanofibers can be integrated into random nanofiber mats, aligned arrays, sandwich devices, stacked or multilayer structures, core–shell nanofibers, textile/yarn/fabric structures, and asymmetric wound-dressing architectures.

These designs can support wearable pressure sensors, tactile sensors, pulse sensors, and respiration sensors, to name a few applications. Additionally, electrospun PVDF-based nanofibers are increasingly being explored for wound-healing and wearable therapeutic applications as they are required to form conformal, biocompatible, mechanically compliant interfaces that need to maintain constant contact with soft tissue while supporting healing, protection, and active stimulation. PVDF-based wound-dressing applications need to be evaluated in terms of materials/device as well as biological metrics, including piezoelectric output, water vapor transmission rate, flexibility, antibacterial activity, cytocompatibility, wound closure rate, inflammatory response, angiogenesis, collagen deposition, re-epithelialization, and tissue regeneration.

Table 7 compares device structures in terms of typical design, functional characteristics, and relevance to pressure sensing, strain sensing, energy harvesting, textile integration, and therapeutic wound-healing bioelectronics.

These device architectures presented in Table 7 represent different engineering tradeoffs rather than a hierarchy of performance. Aligned nanofiber arrays can improve anisotropic load transfer and reduce cancellation of piezoelectric contributions when deformation is preferentially applied along the fiber orientation, whereas randomly oriented mats provide greater multidirectional compliance and porosity but distribute mechanical strain across fibers with different orientations [16,21,45]. Sandwich architectures favor through-thickness stress transfer and electrode charge collection and are therefore well suited to compression-dominant pressure sensing; however, their measured sensitivity can also depend strongly on active-layer thickness, electrode stiffness, encapsulation, contact area, and applied preload [19,21,24]. Stacked and multilayer architectures can increase active piezoelectric volume and enable functional-layer integration, although increased thickness and additional interfaces can alter mechanical compliance and stress distribution [19,20]. Core–shell and composite architectures can improve interfacial coupling, dielectric response, and charge transport; however, excessive filler loading or structural complexity may introduce aggregation, electrical leakage, fiber defects, or reduced flexibility [19]. Textile and fabric-integrated architectures offer advantages in breathability, conformability, and garment-level integration, but introduce different contact mechanics, fiber–textile coupling, durability, and long-term mechanical durability and loading conditions compared to planar sandwich devices [21,34]. Asymmetric multilayer architectures developed for therapeutic applications additionally require simultaneous control of mechanical compliance, tissue contact, moisture management, biocompatibility, sterilization compatibility, and residual-solvent effects. Consequently, increases in β-phase fraction or fiber alignment cannot be considered independently responsible for improvements in voltage output or sensitivity when device geometry, thickness, electrode configuration, mechanical compliance, loading magnitude, frequency, and measurement mode vary simultaneously. Reported performance gains across studies need to be interpreted within their respective test protocols rather than as directly comparable quantitative improvements [19,21].

### 5.3. Wearable Sensing Applications

Electrospun PVDF- and P(VDF-TrFE)-based nanofibers are widely investigated for wearable sensing because their piezoelectric response enables mechanical inputs such as pressure, strain, bending, vibration, body motion, pulse waves, respiration, and chest-wall vibrations to be converted into measurable electrical signals. Their high porosity, mechanical flexibility, and conformability further support integration with skin-mounted and textile-based wearable interfaces. The following subsections examine representative applications in pressure and strain sensing, motion and physiological monitoring, and therapeutic bioelectronics.

#### 5.3.1. Pressure and Strain Sensing and Wearable Interfaces

**Figure 6:**
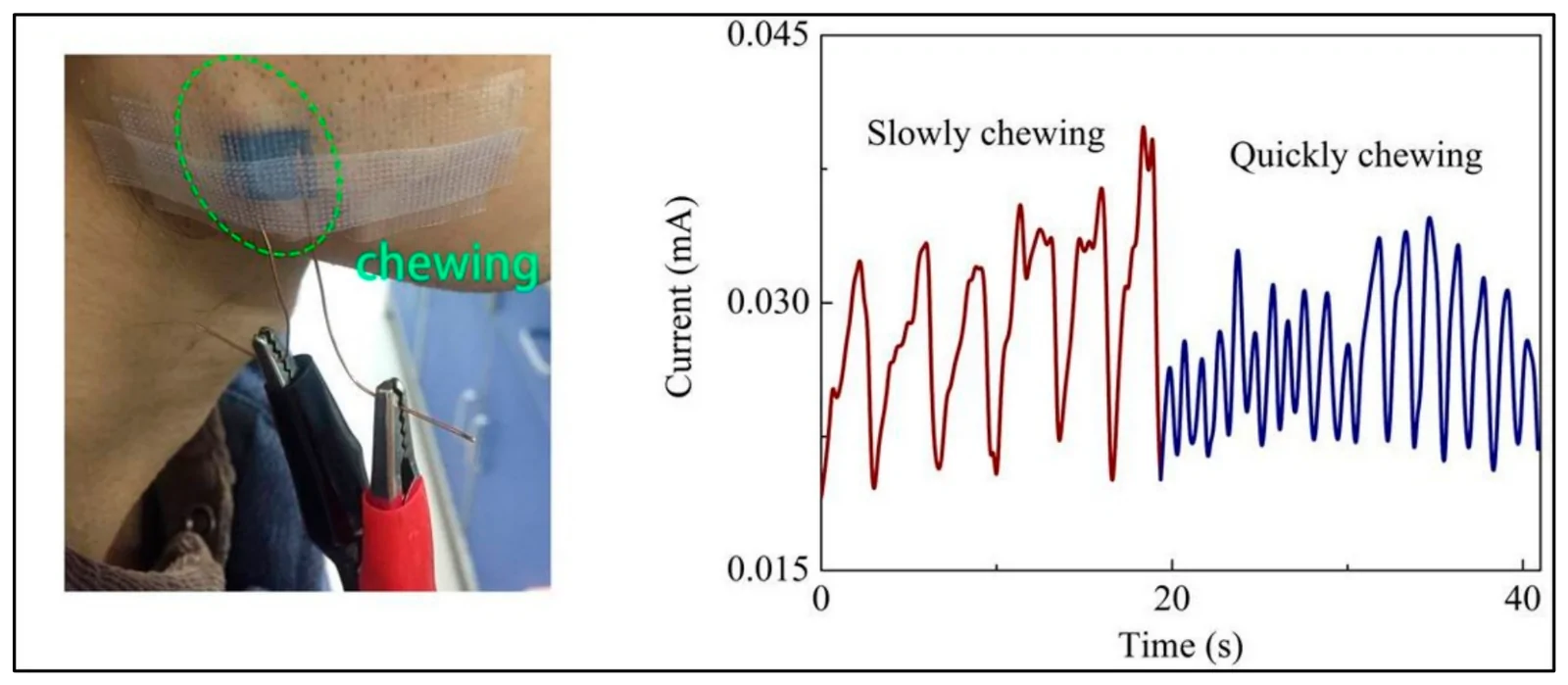
Electrospun PVDF-based wearable sensing platform based on a PEDOT-coated PVDF nanofiber woven fabric (PPNWF) pressure sensor, showing facial muscle movement monitoring. Reproduced from Zhou et al. [34].

Persano et al. demonstrated high-performance self-powered flexible piezoelectric pressure sensor devices based on aligned electrospun P(VDF-TrFE) nanofiber arrays [17]. An electrospun PVDF-GO nanofiber-based self-powered wearable pressure sensor and pyroelectric breathing sensor, developed by Roy et al., demonstrated that interfacial modification and nanofiller incorporation support multifunctional wearable sensing and health monitoring [40]. PEDOT-coated PVDF nanofiber woven fabric (PPNWF) has been reported as a flexible, self-powered electronic-skin platform for health monitoring, motion detection, and muscle tremor detection. In that study, the woven PVDF nanofiber fabric structure helped enhance pressure sensitivity and enabled self-powered sensing behavior [34]. The sensor integrates nanofiber morphology, conductive coating, textile-like architecture, and self-powered sensing capabilities to support health monitoring and human-motion detection, as illustrated in Figure 6.

#### 5.3.2. Motion, Vibration, and Physiological Signal Monitoring

Electrospun PVDF nanofibers are promising candidates for motion and vibration sensing because they respond to dynamic mechanical deformation inputs. For cardiac monitoring, this is especially relevant for seismocardiographic (mechanical) sensing. A conformal, flexible piezoelectric nanofiber sensor can capture the low-amplitude chest wall vibrations associated with cardiac mechanical activity through mechanical coupling to the skin. Electrospun PVDF nanofibers form a class of soft, conformal, flexible, autonomous mechanical sensors that can be easily integrated with wearable cardiac monitoring platforms, offering an interesting alternative to the rigid MEMS accelerometers currently in use. Aligned nanofiber structures with improved anisotropic response and mechanical load transfer are especially useful for vibration and directional mechanical (deformation) sensing, especially compared to their random-fiber counterparts [17,21].

In skin-contact sensors, small fluctuations in body-surface temperature, intermittent thermal contact, expiration of warm exhaled air, or minor local temperature fluctuations may contribute to changes in measured signal values that are otherwise attributed to pressure, pulse, or respiration. Temperature-induced electrical output has previously been shown to produce measurement errors in PVDF pressure sensors, thereby emphasizing that thermal and mechanical contributions should be considered separately when interpreting physiological signals [46]. Therefore, an important consideration in PVDF-based wearable sensing is pyroelectric cross-sensitivity. As polar PVDF exhibits both piezoelectric and pyroelectric behavior, a change in temperature can alter spontaneous polarization and generate electrical charge even in the absence of an externally applied mechanical load. The pyroelectric contribution is transient and depends on the rate of temperature change, whereas the piezoelectric response originates from mechanical deformation.

It is possible to distinguish pyroelectric and piezoelectric contributions experimentally by independently controlling the thermal and mechanical stimuli. Piezoelectric calibration can be performed under approximately isothermal conditions using controlled mechanical loading, whereas the pyroelectric response can be characterized using controlled temperature variation with zero mechanical deformation. The different temporal responses of the two mechanisms can assist signal separation due to slower responses observed with temperature-induced polarization compared to the mechanically induced signals [47].

In multimodal PVDF sensors, additional approaches may include simultaneous temperature monitoring using a reference channel, response-time- or waveform-based decoupling, and device architectures that can be used to generate distinguishable thermal and mechanical signal components. However, temporal separation using a reference channel may not suffice for respiration monitoring applications, as mechanical chest motion/vibration and respiratory temperature fluctuations occur at similar frequencies, requiring independent thermal characterization or an auxiliary temperature channel [47].

Pyroelectricity can also be intentionally exploited rather than treated solely as interference. Roy et al. demonstrated an electrospun PVDF/GO device that functioned as both a piezoelectric pressure sensor and a pyroelectric respiration sensor. The device exhibited a pressure sensitivity of 4.3 V kPa^−1^ under mechanical loading and a maximum pyroelectric power density of approximately 1.2 nW m^−2^ under repeated temperature fluctuations, enabling detection of respiration-induced temperature changes [40].

Thus, it is important to distinguish whether the pyroelectric response represents useful sensing information or unwanted cross-sensitivity based on the physiological variable and sensing methodology, which need to be explicitly identified during device characterization.

#### 5.3.3. Wound-Healing and Wearable Therapeutic Applications

Most wounds involve disruption of the epidermal epithelial layers. Re-epithelialization, which is fundamental to epithelial wound healing and functional tissue regeneration, involves galvanotaxis (cell migration), proliferation, angiogenesis, and differentiation directed by endogenous in vivo electrical fields (EFs) [48,49]. Electrotherapy has been developed as an external stimulation mechanism to augment the endogenous EFs, promoting regeneration and repair. Wound-healing applications are an emerging extension of electrospun PVDF wearable bioelectronics as therapeutic interfaces, especially compared to their conventional electrical stimulation device counterparts, due to their fibrous morphology, flexibility, lightweight structure, and, most importantly, the piezoelectric response. By converting body motion, external vibration, or mechanical deformation into a localized electrical stimulation (piezoelectric potential) signal at the wound interface, piezoelectric dressings tend to supplement the epithelial wound healing electrical microenvironment [50]. Additionally, the electrospun nanofiber morphology can mimic certain characteristics of the extracellular matrix by providing high surface area and breathability. Incorporating functional additives or fillers, such as silver nanoparticles, metal–organic framework particles, magnetic particles, or other bioactive components, can further augment antimicrobial activity, piezoelectric output, magnetoelectric stimulation, and overall wound-microenvironment control.

P(VDF-TrFE) has also been explored for wound-healing applications because its ferroelectric and piezoelectric properties can provide a controllable surface potential and mechanical-to-electrical stimulation. Shi et al. fabricated electrospun P(VDF-TrFE)/BaTiO_3_ membranes that converted ultrasound energy into electrical output and accelerated wound closure under ultrasonic stimulation [51]. The P(VDF-TrFE)/BaTiO_3_ membrane generated up to 8.22 V through 4.5 cm of porcine tissue, while ultrasound-assisted stimulation produced a fibroblast migration rate of 92.6% after 24 h. In a full-thickness dorsal skin-wound model in mice, the P(VDF-TrFE)/BaTiO_3_ membrane under ultrasound stimulation achieved complete wound closure by Day 9, compared with approximately 64% closure in the untreated control group, demonstrating that the ultrasound-induced piezoelectric response could accelerate tissue repair [51]. Additionally, polarized P(VDF-TrFE) films with controllable surface potential and piezoelectric response are able to promote fibroblast proliferation, macrophage polarization, and skin wound healing [52].

Recent studies have reported PVDF-based piezoelectric and electroactive dressings for infected, chronic, and high-mobility wound healing. Chai et al. have developed electrospun ZIF-8/PVDF piezoelectric fiber dressings for infected wound repair, where 5 wt % ZIF-8 nanoparticle incorporation and electrospinning-induced polarization enhanced β-phase formation from 70% to 80.2% and electrical output of the fibers to 2.97 V and 13.7 nA [53]. In a *Staphylococcus aureus*-infected full-thickness skin-defect model in Sprague–Dawley rats, the 5 wt % ZIF-8/PVDF dressing under ultrasound stimulation achieved complete wound closure by Day 14, accompanied by improved granulation-tissue organization, collagen deposition, hair-follicle regeneration, and angiogenic response [53].

**Figure 7:**
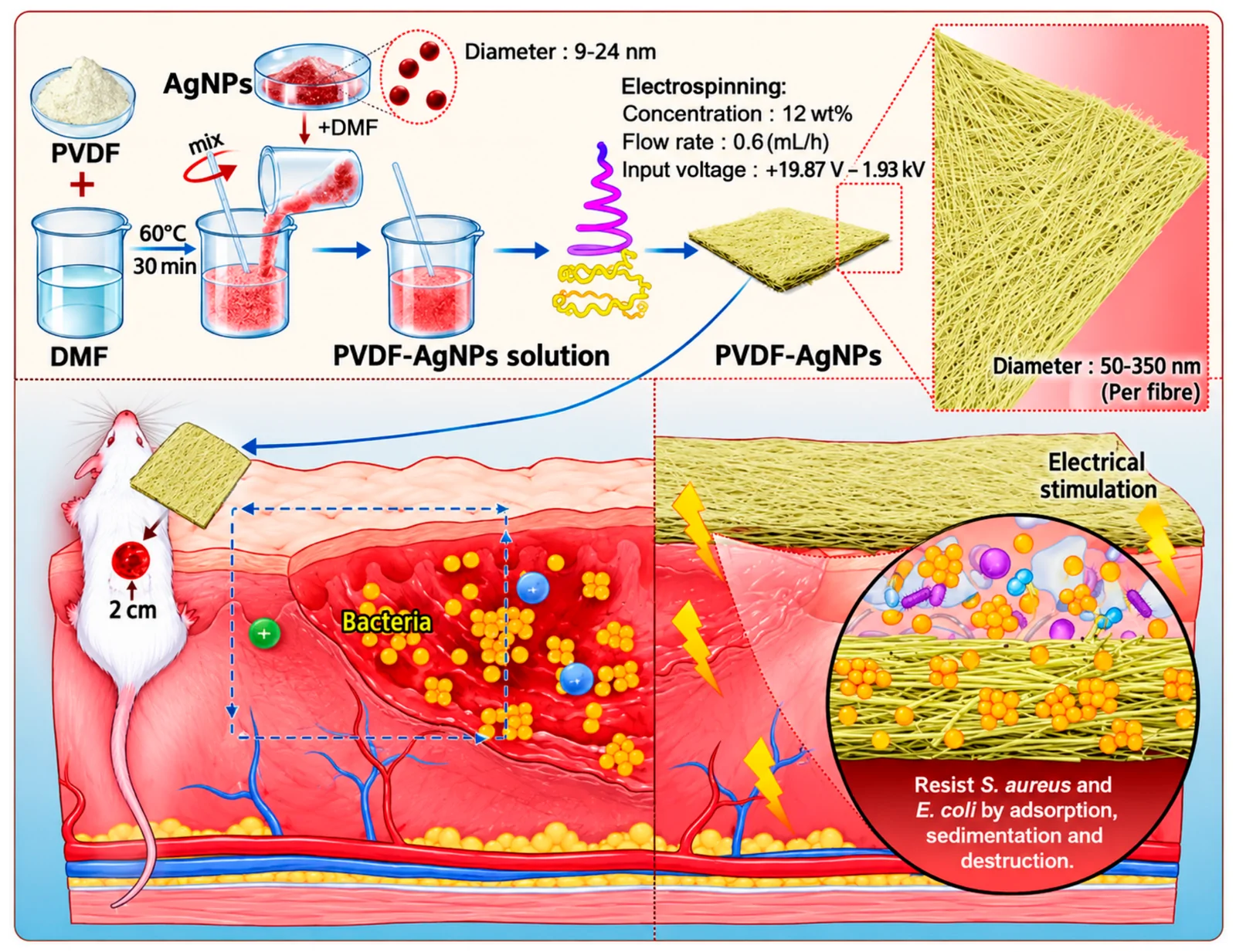
PVDF-AgNP micro-electro nanofibrous dressing for wound-healing applications. Representative electrospun PVDF-based piezoelectric wound dressing incorporating silver nanoparticles for antimicrobial function and mechanically activated electrical stimulation. The PVDF-AgNP nanofibrous dressing is designed for active or high-mobility wound regions, where mechanical deformation can support localized piezoelectric stimulation while maintaining antibacterial activity and wound-healing function. Reproduced from Liu et al. [54].

Liu et al. developed PVDF–AgNP micro-electro nanofibrous dressings for active wound regions, combining PVDF piezoelectric behavior with silver nanoparticle-based antimicrobial function for mechanically active or joint-associated wounds, as shown in Figure 7 [54].

In an evaluation of the wound healing (closure) response in a *Staphylococcus aureus*-infected rat wound model, it was reported that by Day 12, the PVDF-AgNP group showed 93.57 ± 5.07% compared to 39.44 ± 12.74% for the untreated control, 69.52 ± 6.26% for PVDF alone, and 67.09 ± 9.99% for AgNP treatment [54]. Additionally, the PVDF-AgNP dressing substantially reduced inflammatory signaling; TNF-α and IL-6 levels were found to be 315.13 ± 75.97 and 179.78 ± 41.01 pg/mL, respectively, compared to 1414.54 ± 142.16 and 336.84 ± 52.29 pg/mL for PVDF alone [54].

Magnetoelectric composite dressings, such as electrospun CoFe_2_O_4_@CTAB/PVDF dressings for chronic wound healing by coupling magnetic-field-induced stimulation with PVDF-based electroactive behavior, have also shown great promise [55]. As discussed previously, incorporation of functional additives augments wound healing, and piezoelectric additives including barium titanate (BaTiO_3_) and zinc oxide (ZnO) are important inorganic piezoelectric additives in this area. BaTiO_3_ has been incorporated into P(VDF-TrFE) membranes to increase ultrasound-responsive piezoelectric output for wound-healing stimulation [51].

ZnO has been used in PVDF-based wound-healing systems because it can contribute piezoelectric activity and antimicrobial behavior; examples include ZnO-modified PVDF/sodium alginate scaffolds for scar-prevention wound healing and ZnO@PVDF/chitosan nanofibrous membranes for infected wound repair under low-intensity pulsed ultrasound piezoelectric stimulation [56,57].

A 3D-printed ZnO/PVDF/sodium-alginate piezoelectric scaffold containing 0.5 wt% ZnO nanoparticles was evaluated in a full-thickness dorsal wound model in Sprague–Dawley rats and found to have achieved 98.74 ± 0.87% wound closure by Day 14 and antibacterial rates of approximately 99.8% against *E. coli* and 99.93% against *S. aureus*. The scaffold was also found to generate stress-responsive surface microcurrents (0.39 µA initially) that decreased approximately to 0.12 µA as the wound approached closure by Day 14 [56].

Functionally and structurally asymmetric Janus nanofiber architecture-based PVDF dressings represent another important structural direction for piezoelectric wound healing membranes. Hu et al. developed an ultrathin flexible Janus nanofibrous dressing composed of a hydrophilic PCL/Gel layer and a hydrophobic aligned PVDF/Ag piezoelectric layer, allowing unidirectional wound-exudate transport, antibacterial activity, and piezoelectric stimulation under body motion [44]. Along with antibacterial inhibition rates of 99.69% against *E. coli* and 99.84% against *S. aureus*, the open-circuit voltage and the short-circuit current were observed to increase from 1.49 to 5.28 V and 13.76 to 49.36 nA, respectively, under mechanical loading from 0.1 to 1 N in a 10 mm full-thickness *S. aureus*-infected wound model in mice. Normal body motion was found to generate approximately 0.2–1.6 V, and the wounds were found to have almost healed by Day 11, with histological re-epithelialization [44].

Liu et al. reported a gradient porous PVDF/nano-silver (NS25) wound dressing with an asymmetric pore structure (nanoporous top layer (~0.4 µm pores) and a microporous bottom layer (~1.8 µm pores)), as well as antibacterial and antifouling activity. Additionally, the dressing exhibited cytocompatibility, an optimal water vapor transmission rate (~2500 g/m^2^ day) and improved wound closure in vivo [58]. Evaluated in an *A. baumannii*-infected murine full-thickness skin-defect model, the PVDF/NS25 wound dressing was found to achieve approximately 85% wound closure by Day 7, compared to 67.1% for Vaseline gauze and 62.4% for pristine PVDF, respectively, and additionally achieved a faster mean wound-closure time of approximately 9.9 days versus 13.2 days and 13.4 days for Vaseline gauze and pristine PVDF, respectively [58].

Taken together, these studies demonstrate that the combined piezoelectric, antimicrobial and other bio-active functions are able to produce a measurable improvement in infected-wound repair rather than a mere qualitative healing response.

#### 5.3.4. Solvent Selection for Wearable Therapeutic Applications

For skin-contact and wound-healing applications, solvent selection must also be considered from a biocompatibility and post-processing perspective. Polar aprotic solvents such as DMF, DMAc, DMSO, and NMP are widely used because of their ability to dissolve PVDF and their influence on electrospinning stability and electroactive-phase development [59]. However, incomplete solvent evaporation can leave residual solvent within the porous nanofiber membrane, which may contribute to cytotoxicity and confound subsequent biological testing [60]. This concern is particularly relevant for DMF, DMAc, and NMP, which are classified as Class 2 residual solvents under ICH Q3C, whereas DMSO is classified as Class 3 [61]. Therefore, controlled removal and analytical verification of residual solvents in biomaterial processing are essential.

Residual-solvent control should therefore be incorporated into the fabrication workflow through sufficient evaporation followed by vacuum drying or other validated post-processing, with residual solvent quantified where possible, for example by headspace gas chromatography, before cytocompatibility or skin-contact evaluation. This is especially important because solvent volatility also influences PVDF fiber morphology and β-phase formation; consequently, simply replacing a high-boiling solvent with a more volatile system may change both residual-solvent burden and piezoelectric performance. Studies of electrospun PVDF have shown that DMF-, DMSO-, NMP-, and DMAc-based solvent systems can produce substantially different β-phase fractions and fiber morphologies, demonstrating that biocompatibility-driven solvent substitution must be balanced against electroactive-phase control [13].

Sterilization requires critical consideration. Electrospun PVDF-based wound dressings may be sterilized using ethylene oxide (EtO), ionizing radiation, or other low-temperature approaches; however, sterilization should not be assumed to be structurally or electrically neutral. EtO avoids the high temperatures associated with steam sterilization, but retained EtO and its by-products require adequate post-sterilization aeration, particularly in highly porous fibrous matrices. Ionizing radiation can induce chain scission, crosslinking, and changes in crystallinity; in polarized PVDF films, irradiation has been shown to preserve the piezoelectric response while modifying β-phase crystallinity at lower irradiation doses. Thermal or steam exposure may likewise alter polymer morphology or crystalline-phase distribution because PVDF phase composition is sensitive to thermal history. Therefore, sterilization protocols for wearable piezoelectric dressings should be validated not only for sterility but also for their effects on fiber morphology, β-phase fraction, mechanical integrity, and piezoelectric output. Pre- and post-sterilization characterization by FTIR/XRD together with electrical or piezoelectric measurements would provide a more rigorous basis for evaluating device performance after sterilization [62].

### 5.4. Energy-Harvesting Applications

Rapid development of passive and low-power portable and wearable electronics has increased interest in sustainable power sources capable of converting ambient or human-generated mechanical energy into electrical energy. In the emerging field of nanoenergy, mechanical energy harvesting offers a potential means of supplementing or reducing dependence on conventional batteries in miniaturized wearable systems [63]. A device can be classified as an energy harvester when it is able to transduce and convert ambient energy or human kinetic energy into electrical energy. An energy-harvesting system, on the other hand refers to a complete system architecture integrating a mechanical transducer (nanogenerator), impedance-matching networks, AC–DC rectification, power management circuitry, and energy storage elements. In this case, the harvested power equals or exceeds the total system load demand as well as any internal circuit overhead, allowing it to autonomously drive external active modules such as data acquisition, communication and so on for dynamic sensing applications [64].

Nanogenerators are currently being investigated widely as alternative power sources (to batteries) for autonomous self-powered applications [65]. A nanogenerator represents a micro- or nano-scale electromechanical transducer element that is able to convert localized mechanical energy into raw, unrectified electrical signals (current and voltage) using piezoelectric, triboelectric or pyroelectric mechanisms without inherently integrated power management or energy storage [63,65]. Piezoelectric nanogenerators (PENG) and triboelectric nanogenerators (TENG) rely on mechanical deformation-induced polarization changes and contact electrification/electrostatic induction, respectively, whereas pyroelectric nanogenerators generate electrical output through temperature-change-induced variations in the spontaneous polarization of a pyroelectric material [66].

Electrospun PVDF nanofibers are widely used in piezoelectric nanogenerator applications because they can convert repeated mechanical deformation into electrical output. In a typical PVDF-based piezoelectric nanogenerator, the nanofiber mat is placed between electrodes, and when the device is compressed, bent, stretched, or vibrated, the electroactive PVDF domains undergo polarization changes that induce charge accumulation at the electrodes, producing voltage and current output.

Fang et al. demonstrated an electrical power generator based on randomly oriented electrospun PVDF nanofiber membranes, showing that even non-aligned electrospun PVDF mats can be used for mechanical energy conversion [45]. Chang et al. reported a direct-write piezoelectric polymeric nanogenerator for flexible energy conversion [11], establishing electrospun and printed PVDF-based architectures for lightweight mechanical energy harvesters. Table 8 summarizes energy-harvesting performance, distinguishing absolute power from areal and volumetric power density, as reported in the literature.

A major advantage of PVDF-based energy harvesters is that the same piezoelectric material can function as both a sensor and a power-generating element. Electrospun PVDF-based nanofiber layers can serve as flexible mechanical sensors for pressure, vibration, body motion, and physiological mechanical signals, while related PVDF-based piezoelectric nanogenerator structures can harvest mechanical energy from body movement [21].

A hybrid PENG/TENG device combines both piezoelectric and triboelectric transduction mechanisms within a unified structural footprint to exploit their complementary current-voltage characteristics and frequency responses [63,65]. In a PENG, mechanical deformation changes polarization within the piezoelectric layer, whereas a TENG generates charge primarily through contact electrification coupled with electrostatic induction. Combining these mechanisms can broaden the range of mechanical inputs that contribute to electrical output and, depending on device architecture and loading conditions, can increase output voltage, transferred charge, power density or sensing sensitivity compared to a single-mode device. Hybrid PENG/TENG systems may therefore provide improved mechanical-energy conversion across varied biomechanical inputs and operating frequencies [69].

Chen et al. developed a stretchable PVDF–PDMS hybrid piezoelectric–triboelectric nanogenerator in which electrospun PVDF nanofibers were embedded at the PDMS surface, enabling simultaneous piezoelectric and triboelectric transduction [70]. Under controlled testing at 1 Hz and an applied force of 10 N, the hybrid device produced higher electrical output than the corresponding single-mode PENG and TENG configurations. Beyond peak electrical output, the device maintained its layered structure after 5000 pressure-loading cycles and was able to charge a 1.0 μF capacitor to 3 V within 90 s, as well as demonstrating sufficient output capable of powering ten LEDs. These results illustrate that hybridization can improve not only instantaneous electrical response but also practical energy-storage capability, although the usable system-level power remains dependent on loading conditions, impedance matching, rectification, and energy storage circuitry [70].

The multimodal electrospun PVDF–graphene oxide (GO) nanofiber system (Table 8) illustrates a slightly different form of hybrid functionality in which the same electrospun active layer responds to both mechanical and thermal stimuli. Mechanical pressure, bending, stretching, and vocal vibration are detected primarily through the piezoelectric response, whereas respiration-induced temperature fluctuations generate a pyroelectric signal. Roy et al. reported a pressure sensitivity of 4.3 V/kPa with detection down to 10 Pa and a maximum piezoelectric power density of approximately 6.2 mW/m^2^ under compressive loading. In comparison, the maximum pyroelectric power density was approximately 1.2 nW/m^2^ under repeated temperature fluctuations, indicating that the thermal channel is more relevant as a sensing modality than as a significant power source. Thus, the piezoelectric and pyroelectric outputs arise from different physical inputs and contribute to substantially different power levels [40].

Energy-autonomous operation represents a more stringent system-level condition in which the harvested and stored energy is sufficient to support the total power demand of active sensing, processing, and wireless communication modules over the required operating cycle. By contrast, a self-powered sensor functions as an active transducer in which the stimulus-generated electrical output, expressed as voltage, current, or accumulated charge, serves directly as the analytical sensing signal without requiring an externally applied excitation voltage at the transducing element [54]. This architecture is particularly attractive for wearable devices where device footprint, rigidity, battery size, lifetime, conformability, and comfort are important constraints. However, it is important to note that in a self-powered sensor, the downstream signal-conditioning, processing, or wireless-communication electronics are not necessarily powered by the generated signal.

An example of this transduction approach includes self-powered wearable sensors based on aligned P(VDF-TrFE)/MWCNT composite nanofibers developed for physiological and muscular motion monitoring. Mechanical stretching of the electrospun composite produced an aligned membrane with a reported *d*_33_ of approximately 50 pm/V and a force sensitivity of approximately 540 mV/N. Under periodic compression, the sensor generated approximately 5 V at 2.5 N and 0.6 Hz, with no obvious voltage degradation during 3.5 h of repeated loading. The same device was able to detect weak physiological and muscular signals, producing approximately 0.1 V, 0.75 V, 0.3 V, and up to 1.8 V during breathing, swallowing, chewing, and finger movement, respectively [37].

In wearable bioelectronics, electrospun PVDF nanofibers may be particularly useful for both functional sensing and mechanical energy harvesting when integrated with flexible electrodes, conformal data-acquisition and signal-conditioning electronics, wireless data-transfer modules, and algorithm-enabled analysis to support autonomous or semi-autonomous wearable platforms. System-level integration is illustrated by the recent electrospun P(VDF-TrFE) cardiovascular-monitoring platform reported by Zhang et al. through integration of a nanofibrous piezoelectric sensor with flexible CNT electrodes, PDMS encapsulation, and a 25 μm-thick polyimide flexible PCB containing on-body charge-amplification and filtering circuitry, enabling acquisition of radial, carotid, and seismocardiogram signals from the wrist, neck, and chest, respectively [39]. Thermal annealing increased the β-phase fraction from 73% to 81% and overall crystallinity from 74.1% to 84.7%, accompanied by an approximately 40% increase in peak-to-peak voltage and an improvement in force sensitivity from 47.6 to 101.2 mV/N. The annealed sensor exhibited a linear force response over 5–25 N at 1 Hz and maintained stable output through 10,000 loading cycles at 5 N and 1 Hz, as well as 5000 bending cycles with negligible loss in piezoelectric response. This system demonstrates that improvements in material-level piezoelectric properties can translate into greater device sensitivity; however, practical wearable performance additionally depends on mechanical durability, conformal electrode integration, high-impedance signal conditioning, and integration of the sensing element with body-worn electronics.

## 6. Challenges and Future Perspectives

Despite substantial progress in electrospun PVDF-based wearable bioelectronics, several challenges continue to separate material-level demonstrations from reliable wearable systems. A central challenge is fabrication reproducibility. Electrospinning remains sensitive to precursor composition, polymer molecular weight and concentration, solvent system, applied voltage, flow rate, needle-to-collector distance, collector design, humidity, and temperature, with relatively small variations capable of altering fiber morphology, alignment, crystallinity, electroactive-phase content, and ultimately electromechanical response [13,21]. Future studies should therefore move beyond optimization of individual parameters toward improved reporting and control of the complete processing window, including environmental conditions, precursor preparation, post-processing, and batch-to-batch variability. For composite systems, this requirement also extends to filler concentration, dispersion, interfacial compatibility, and stability. Emerging fillers such as MXenes provide opportunities to tailor interfacial polarization, electroactive-phase development, dielectric response, and electrical properties; however, these advantages must be balanced against aggregation, oxidation, restacking, electrical leakage, altered mechanical compliance, and processing variability.

Establishing meaningful relationships between material characterization and device-level performance remains another major challenge. Crystallinity, electroactive-phase content, dipole organization, fiber alignment, and piezoelectric coefficients provide important information about the electromechanical behavior of PVDF-based materials, while practical device performance additionally depends on active-layer thickness and area, electrode configuration, mechanical coupling, loading direction and frequency, encapsulation, device architecture, and measurement conditions. Cross-study comparisons should therefore consider the material composition, structural characterization, device geometry, loading protocol, and measurement basis together. Future studies would benefit from reporting clearly defined material- and device-level characterization alongside realistic operating conditions so that processing, structure, electromechanical properties, and wearable performance can be interpreted within a consistent process–structure–property–performance framework.

Wearable validation must also extend beyond controlled benchtop loading. Physiological mechanical inputs are generally low-amplitude, low-frequency, multidirectional, and dependent on anatomical location and activity. Skin deformation, perspiration, clothing, sensor placement, attachment pressure, motion artifacts, and repeated flexing can further modify sensor response. These considerations are particularly important for low-amplitude physiological mechanical signals such as pulse and SCG signals, for which conformal contact, sensor placement, mechanical coupling, and signal-to-noise ratio can be as important as the material-level piezoelectric response. Long-duration testing under realistic body motion, cyclic deformation, humidity, temperature variation, and repeated attachment is therefore important for evaluating practical wearable operation. For therapeutic and wound-interface systems, translation additionally requires consideration of residual solvent, sterilization compatibility, moisture management, biocompatibility, long-term tissue contact, and preservation of material and electromechanical properties following sterilization and storage.

Mechanical energy harvesting has been extensively investigated as a route toward self-powered electronic systems, including wearable and implantable biomedical devices. Mechanical energy represents an abundant but largely dissipated energy source in everyday environments and can be converted into electrical energy through mechanisms including electromagnetic induction, magnetostriction, piezoelectricity, triboelectricity, and electrowetting [69]. However, several challenges, such as the ability to generate sufficient and stable electrical output, withstand prolonged and repeated mechanical loading, support scalable and cost-effective fabrication, and remain responsive to relatively small and irregular mechanical inputs, such as those generated by human motion, continue to limit their practical implementation [69]. No single energy-harvesting mechanism currently satisfies all of these requirements across wearable applications, emphasizing the need for application-specific material-, device-, and system-level optimizations.

Practical energy harvesting from body motion remains challenging because physiological mechanical inputs are often low-frequency, low-amplitude, and variable across body locations and activities [71]. Future work should focus on evaluation of energy-harvesting output at realistic mechanical inputs, including repeated bending, walking, respiration, chest-wall vibration, and other representative body motions, rather than relying exclusively on controlled laboratory excitations, as well as on system-level integration. Such evaluation should include electrical output under representative loading conditions together with integration of power-management, energy-storage, and voltage-regulation modules to establish practical energy-harvesting capability. Additionally, translation of electrospun PVDF piezoelectric systems from laboratory demonstrations to practical wearable technologies further requires coordinated optimization of mechanical coupling, electrode configuration, load resistance, encapsulation, body placement, and system integration to convert material-level piezoelectric response into stable and usable electrical output [17,19]. Based on these limitations, the most realistic near-term role of electrospun PVDF energy harvesters may be as self-powered sensing elements or auxiliary energy sources rather than complete replacements for batteries in wearable healthcare systems [16].

Hybrid PENG/TENG architectures provide another route toward expanding mechanical-energy conversion by combining piezoelectric and triboelectric mechanisms within a single system. Such hybridization can broaden the range of mechanical interactions contributing to electrical output; however, reported improvements must be interpreted in relation to device area, architecture, loading magnitude and frequency, electrical configuration, and the dimensional basis used for voltage, current, and power measurements. Hybridization should therefore be evaluated as an application-specific system-design strategy rather than assumed to provide intrinsically superior energy-harvesting performance.

System-level advancements should focus on coordinated optimization across materials, device architecture, electronics integration, manufacturing, and validation to enable highly specialized and accurate application-specific platforms. These target-optimized systems should be designed around the relevant mechanical stimulus, such as pressure, strain, pulse, vibration, or motion, while also accommodating application-specific requirements including fiber alignment, membrane thickness, electrode configuration, and electromechanical coupling strategies. Multimodal wearable platforms should additionally integrate ML-enabled post-processing and classification of raw sensor data to support more comprehensive interpretation of physiological signals and their broader health implications.

Artificial intelligence (AI) and machine-learning (ML) approaches are increasingly being integrated with wearable physiological sensing systems to extract clinically or physiologically relevant information from continuous, multidimensional biosignals [72,73]. In particular, ML has been applied to feature extraction, signal classification, artifact management, personalized monitoring, and multimodal fusion of signals such as electrocardiogram (ECG), seismocardiogram (SCG), and photoplethysmogram (PPG) [73,74,75]. Recent wearable cardiac systems have demonstrated ML-based analysis of synchronized ECG and SCG signals for cardiovascular assessment, while larger clinical studies have combined ECG, SCG, and PPG with ML to estimate hemodynamic parameters [74,75]. For electrospun PVDF-based wearable systems, these developments suggest a future pathway in which piezoelectric signals associated with multi-modal sensing involving pulse, respiration, body motion, and SCG are combined with complementary physiological modalities and processed using AI/ML for signal denoising and artifact rejection, automated feature extraction, event and pattern classification, multimodal sensor fusion, subject-specific calibration, and physiological-state assessment. AI/ML can thereby support downstream processing of variable, motion-sensitive, and often low-amplitude wearable signals to improve their reliability and physiological interpretability. This may be particularly valuable for multimodal cardiac monitoring, where mechanical signals such as SCG can be analyzed together with electrical or optical signals such as ECG or PPG to improve temporal feature detection and context-specific interpretation [73,74]. Translation to wearable implementation therefore requires consideration of training-data quality, inter-subject variability, ground-truth labeling, model generalizability, and computational constraints including memory, latency, and energy consumption [73,76]. Recent edge-ML approaches further indicate that denoising, feature extraction, classification, and inference may increasingly be performed locally on resource-constrained wearable or paired devices, reducing dependence on continuous cloud connectivity and potentially improving response time and data privacy. These advantages must nevertheless be balanced against requirements for model compression, computational efficiency, validation across diverse populations and operating conditions, and the added power burden associated with continuous on-device inference [75,76].

**Figure 8:**
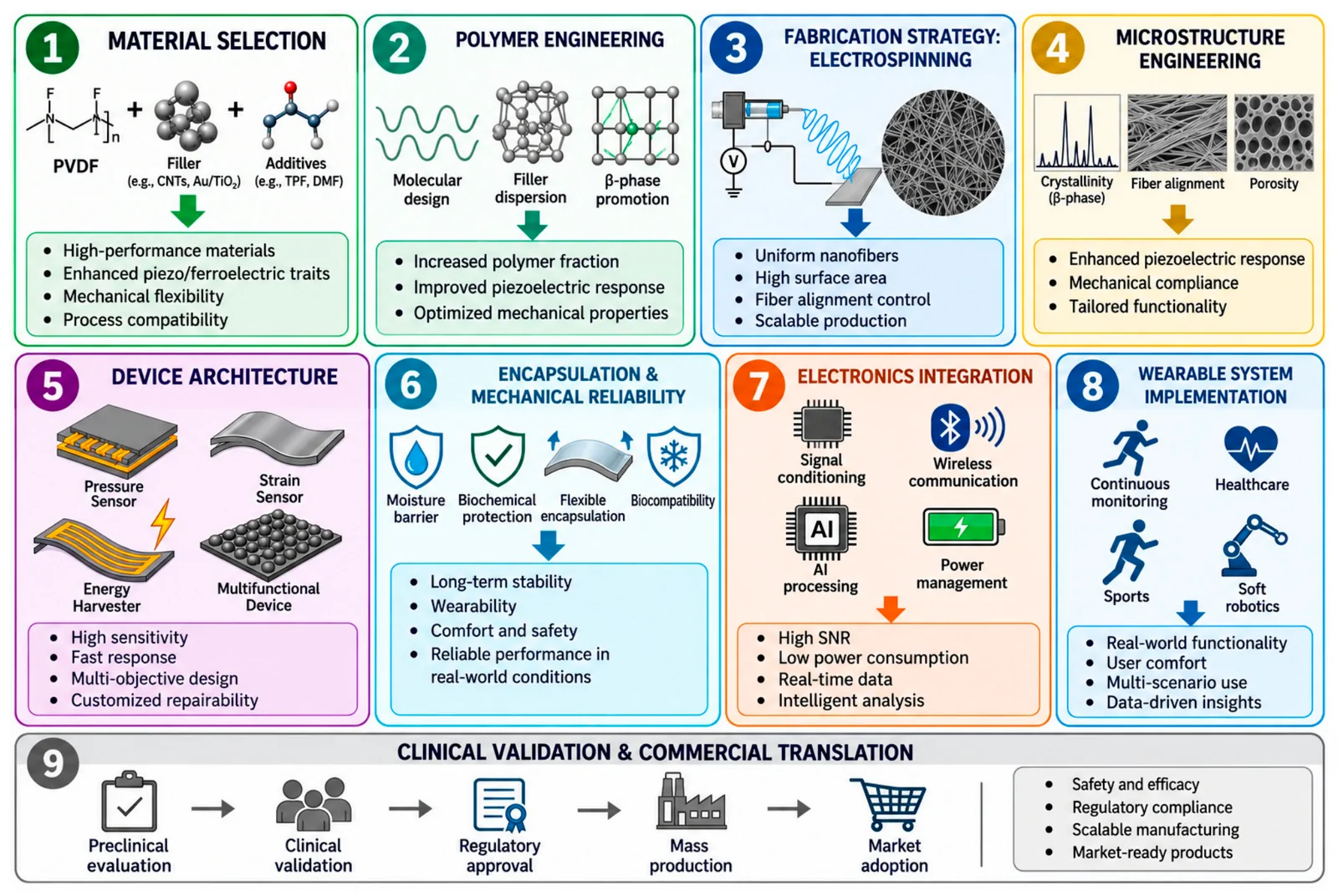
Conceptual engineering roadmap for the development and translation of electrospun PVDF wearable piezoelectric systems. The roadmap integrates material performance, manufacturability, device reliability, biocompatibility, electronics integration, and application-specific validation as interdependent engineering considerations for translation toward scalable wearable bioelectronic technologies.

These interconnected considerations are organized within the conceptual engineering roadmap shown in Figure 8, which links material selection and polymer engineering with electrospinning, microstructure development, device architecture, electronics integration, wearable-system implementation, and eventual clinical or commercial translation. At the material and processing levels, precursor composition and electrospinning conditions govern electroactive-phase formation, fiber morphology, alignment, porosity, crystallinity, and dipole organization. These structural characteristics subsequently influence electromechanical performance, but their practical value depends on effective mechanical coupling, electrode configuration, encapsulation, and device architecture. At the system level, signal-conditioning electronics, wireless communication, power management, mechanical reliability, biocompatibility, manufacturability, and application-specific validation become equally important. The roadmap therefore provides a conceptual framework for organizing the process–structure–property–performance relationships and translation requirements identified throughout this review, rather than representing a validated sequence of technology-readiness stages.

## 7. Conclusions

Electrospun PVDF- and P(VDF-TrFE)-based nanofibers provide a versatile platform for wearable sensing, physiological monitoring, therapeutic interfaces, and mechanical energy conversion. Their performance is governed by an interconnected hierarchy spanning precursor formulation, electrospinning conditions, fiber morphology and alignment, crystalline and dipolar organization, device architecture, electrode configuration, mechanical loading, and system-level integration.

Electrospinning is particularly well suited to wearable bioelectronics as it enables the development of mechanically compliant, porous, and structurally tunable fibrous systems while supporting alignment, composite incorporation, multilayer architectures, and integration with flexible electrodes and textiles. Successful translation of these attributes at device and system levels, however, requires careful understanding and evaluation as well as coordinated optimization of the complete process–structure–property–performance pathway. Practical translation additionally requires manufacturing reproducibility, mechanical reliability, biocompatibility, manufacturability, system integration, and application-specific validation.

Reported system-level performance metrics across studies highlight considerable diversity in material compositions, device configurations, characterization approaches, and mechanical testing conditions. A more unified evaluation framework is therefore required to relate material properties and device architecture to system-level performance under realistic wearable conditions, enabling reproducible structure–property–performance relationships and more standardized reporting across studies.

Future progress will increasingly depend on integration rather than isolated improvements in material output. Composite engineering, including emerging MXene-containing systems, multimodal sensing, edge-AI-enabled signal processing and classification, hybrid energy-harvesting strategies, flexible electronics, and application-specific device architectures can expand the capabilities of electrospun PVDF systems, but also introduce new requirements for reproducibility, mechanical reliability, biocompatibility, manufacturing scalability, and system validation. Addressing these requirements will determine whether electrospun PVDF-based technologies progress from laboratory demonstrations toward robust wearable sensing, therapeutic, and self-powered bioelectronic systems.

## Figures and Tables

**Table 1:** PVDF crystalline phases, electroactive behavior, and relevant sensing/energy-harvesting applications.

PVDF Phase	Chain Conformation	Dipole Arrangement	Electroactive Behavior	Relevance	Refs.
α	TGTG′	Antiparallel dipoles	Mostly nonpolar	Common default phase; low piezoelectric response	[8,15,16]
β	TTTT	Strong parallel dipole alignment	Strongly electroactive	Most desirable phase for sensing and energy harvesting	[7,8,9,14,15,16,17]
γ	Mixed TG	Partially polar	Moderately electroactive	Can contribute to electroactive behavior depending on processing	[8,15,16]
δ	Polar form related to α-phase	Parallel dipoles after poling	Electroactive	Less commonly targeted; processing-dependent	[8,15]
ε	Related nonpolar phase	Antiparallel dipoles	Weak or nonpolar	Less relevant for wearable piezoelectric devices	[8,15]

**Table 2:** Comparison of common PVDF fabrication methods.

Fabrication Method	PVDF Film Layer	Advantage	Limitation
Solution Casting	Dense film	Simple, low-cost, filler-compatible	Often needs stretching/poling; limited fiber morphology
Spin Coating	Thin film	Uniform films for microdevices	Planar geometry; limited porosity
Melt Processing	Films/sheets	Scalable, solvent-free	Often needs post-processing for β-phase
Phase inversion	Porous membrane	Porous structure, membrane applications	Less chain alignment than electrospinning
Printing/Direct Writing	Patterned layers	Scalable patterned fabrication	Ink formulation and post-poling challenges
Electrospinning	Nanofiber mats/arrays	β-phase enhancement, high surface area, flexibility, alignment control	Parameter-sensitive; scaling and reproducibility challenges

**Table 3:** Comparison of PVDF fabrication strategies for wearable piezoelectric devices.

Fabrication Method	β-Phase (%)	β-Phase Determination	Sample	Direct |*d*_33_| (pC/N)	Converse |*d*_33_| (pm/V)	Ref.
Aligned Electrospinning	88 ± 1	FTIR	Aligned PVDF nanofibers	|27.4 ± 1.5|	—	[28]
Solution Casting	83.7	FTIR-ATR; XRD confirmation	Hot-pressed, thermally poled PVDF film	—	|45|	[29]
Spin Coating	~60	IR spectroscopy; XRD, Raman	PVDF thin film, ~170 nm	—	|74|	[30]
Melt Processing	~31	FTIR; XRD/DSC supporting characterization	Compression-molded PVDF	|9.1 ± 6.9|	—	[31]
Phase Inversion (with Spin Coating)	97.82	FTIR	30 wt.% PVDF, 2500 rpm, liquid-N_2_ quench	—	|29.87|	[32]
Printing/ Direct Writing	60	FTIR; 763/840 cm^−1^ absorbance analysis	Printed and hot-pressed PVDF	|6.42 ± 0.78|	—	[33]

Note: Piezoelectric coefficients are reported as absolute magnitudes, |*d*_33_|, to present the reported values consistently without implying performance differences based on sign. The sign of a reported coefficient depends on the polarization direction, coordinate system, and deformation convention used in the original measurement and therefore is not interpreted here as an indicator of superior or inferior piezoelectric performance.

**Table 4:** Comparative engineering assessment of PVDF fabrication strategies for wearable bioelectronics.

Fabrication Method	Flexibility	Scalability	Process Complexity	Cost	Mfg. Readiness	Ref.
Electrospinning	H	M	H	M	M	[22]
Solution Casting	H	H	L	L	H	[23]
Spin Coating	M	M	M	M	M	[24]
Melt Processing	M	H	M	M	H	[25]
Phase Inversion	H	M	M	M	M	[26]
Printing/Direct Writing	H	H	H	M	M	[27]

Note: H = High; M = Moderate; L = Low. While a higher rating indicates greater capability for Flexibility, Scalability, and Manufacturing Readiness, it indicates a greater processing or resource burden for the Process Complexity and Cost categories.

**Table 5:** Rating framework for qualitative ratings used in Table 4.

Attribute	High	Moderate	Low
Flexibility	Highly compliant/conformal structures suitable for repeated deformation	Flexible structures with limitations from thickness, morphology, substrate, or post-processing	Limited compliance and conformability; substantial modification required for flexible use
Scalability	Compatible with continuous, established high-throughput manufacturing	Scale-up feasibility with throughput, uniformity and process-control limitations	Primarily batch or laboratory-scale, low-throughput processes with barriers to translation
Process Complexity	Specialized equipment with multi-parameter control and/or substantial post-processing	Controlled processing with moderate equipment and/or post-processing requirements	Simple fabrication with few process-control parameters and/or minimal post-processing burden
Cost	High material; specialized equipment, with multiple processing/post-processing steps	Moderate material, equipment and processing economic burden	Low-cost materials/equipment with minimal processing burden
Manufacturing readiness	Established process; readily transferable to mature industrial-scale processing	Credible manufacturing scale-up route with process control, integration and optimization challenges	Predominantly proof-of-concept approach with limited scalable manufacturing scope

**Table 6:** Representative device-level sensing metrics for electrospun PVDF-based wearable systems.

Material/Architecture	Force Sensitivity (V/N)	Pressure Sensitivity (V/kPa)	Strain Sensitivity (V/%)	Output Voltage	Test Condition/ Device Geometry	Wearable Application	Ref.
Electrospun P(VDF-TrFE) nanofiber web	0.0605	—	—	~5 mV	Nanofiber-web force sensor; active area NR; Dynamic response maintained up to 20 Hz; Small applied loads	Textile-based flexible breathable force/pressure sensing	[35]
P(VDF-TrFE) nanofiber mat with 3D PDMS sheet	23 (flat); 20 (wrist); 12 (fingertip)	—	—	~1.0 V flat/wrist; ~0.5 V fingertip	Customized flat, wrist, and fingertip geometries (based on mechanical loading); repeated stable cyclic operation; repeated tactile/mechanical deformation	Tactile sensing and Radial-pulse sensing	[36]
Aligned electrospun PVDF/CNT nanofibrous membrane	0.00226	—	0.00429	1.89 V at 100° bending	4 cm-diameter samples; Cu-foil electrodes; 8 N prestress; 200–350 N compression at 0.5 Hz; stable cyclic compression/tension; dynamic compression and tensile loading	Flexible wearable mechanical sensing	[28]
Stretched aligned P(VDF-TrFE)/MWCNT composite nanofibers	0.540	—	—	5 V at 2.5 N, 0.6 Hz; 3.5 h under repeated 2.5 N loading	Linear-motor compression (0.5–5 N); 2.5 N at 0.6 Hz	Pulse, breath, and small-motion (swallowing, chewing, finger and wrist) sensing	[37]
P(VDF-TrFE)/PDMS matrix with inkjet-printed PEDOT:PSS electrode	0.254	—	—	~0.8 Vpp at 1.25 N, 3 Hz	1 cm^2^ device; 1.25 N impact at 3 Hz; inkjet-printed PEDOT:PSS electrodes (resistance characterized through 30,000 bending cycles)	Radial/carotid pulse rate, breathing and foot-movement sensing	[38]
P(VDF-TrFE) with CNT electrodes and PDMS encapsulation	0.0476 non-annealed; 0.1012 annealed	—	—	Higher Vpp (~40%) after annealing	PDMS/CNT/P(VDF-TrFE)/CNT/PDMS stack; shaker-based testing; >10,000 loading cycles at 5 N, 1 Hz; 5000 bending cycles	Radial, carotid and seismocardiogram monitoring	[39]
Electrospun PVDF/graphene oxide nanofiber	—	4.3	—	7 V at 10 kPa	Flexible PVDF/GO nanofiber device (active area NR); repeated pressure loading; evaluated under bending/thermal variation	Pressure, bending/stretching, vocal vibration and breathing (pyroelectric) sensing	[40]

Note: Force sensitivity (V/N), strain sensitivity (V/%), and pressure sensitivity (V/kPa) describe different response metrics. NR indicates that the parameter was not reported by the source.

**Table 7:** Comparison of electrospun PVDF device architectures and their functional characteristics for wearable bioelectronics.

Device Structure	Typical Design	Device Characteristics	Relevance
Free-standing Mat	Random PVDF nanofiber membrane between electrodes	High deformability and porosity; less directional response	Simple pressure, motion, and energy-harvesting devices
Aligned Nanofiber array	PVDF or P(VDF-TrFE) fibers collected with directional alignment	Improved directional response and anisotropic load transfer	Strain, vibration, and SCG-relevant sensing
Sandwich	Electrode/PVDF nanofiber/electrode	Efficient through-thickness stress transfer and charge collection under compression-dominant loading	Pressure sensors and nanogenerators
Stacked/multilayer	Multiple PVDF or composite PVDF layers	Increases active piezoelectric volume and enables functional-layer design; added thickness can alter compliance and stress distribution	Higher-output sensors and energy harvesters
Core–Shell	Functional core with PVDF shell or PVDF-based core–shell design	Enhances coupling, sensitivity, or charge collection	Advanced wearable pressure sensors
Textile/Yarn/Fabric Structures	PVDF nanofibers integrated into yarns, woven fabric, or textile substrates	Supports breathability, comfort, and garment integration	Wearable sensors and e-textiles
Asymmetric Multilayer	PVDF layer combined with moisture-control, antibacterial, and/or other protective layers	Provides wound management and therapeutic function in addition to piezoelectric stimulation	Wearable wound-healing bioelectronic dressings

**Table 8:** Representative energy-harvesting performance metrics for electrospun PVDF-based wearable systems.

Material/ Architecture	Output Voltage	Absolute Power (µW)	Areal Power Density (W/m^2^)	Volumetric Power Density (W/m^3^)	Test Condition/ Device Geometry	Wearable Application	Ref.
Electrospun PVDF/graphene oxide nanofibers	7 V at 10 kPa	—	0.0062	—	Compressive-pressure loading; repeated pressure/temperature operation;	Multifunctional; pressure, bending/stretching, vocal vibration (piezoelectric) and respiration (pyroelectric) sensing	[40]
Electrospun P(VDF-TrFE) nanofiber with PDMS/MWCNT hybrid nanogenerator	Piezoelectric: 2.5 Vpp; Triboelectric: 25 Vpp	Piezoelectric: 9.74; Triboelectric: 98.56	—	Piezoelectric: 689; Triboelectric: 1980	Repeated electromechanical operation (1–4 Hz); 5 N mechanical loading;	Wearable finger-motion energy harvesting using hybrid PENG/TENG	[67]
Free-standing electrospun P(VDF-TrFE) membrane	18.1 V	—	0.229	—	3.4 V at 25 kPa; 10.6 V at 75 kPa Dynamic testing at 75 kPa over 0.2–2 Hz; 5000 cycles at 75 kPa with ~5% voltage attenuation.	Self-powered biomechanical (foot/elbow/finger) monitoring	[68]

Note: Absolute power, areal power density, and volumetric power density are reported separately because they have different dimensional bases. Unit normalization performed within equivalent dimensions. NR indicates that the parameter was not reported in the original source; missing geometric or loading parameters were not inferred.

## Data Availability

Dataset available on request from the authors.
